# A Precise and GNSS-Free Landing System on Moving Platforms for Rotary-Wing UAVs

**DOI:** 10.3390/s19040886

**Published:** 2019-02-20

**Authors:** Francisco Alarcón, Manuel García, Ivan Maza, Antidio Viguria, Aníbal Ollero

**Affiliations:** 1 Center for Advanced Aerospace Technologies, Calle Wilbur y Orville Wright, 19, La Rinconada, 41300 Sevilla, Spain; falarcon@catec.aero (F.A.); mgarcia@catec.aero (M.G.); aviguria@catec.aero (A.V.); 2 Robotics, Vision and Control Group, University of Seville, Avda. de los Descubrimientos s/n, 41092 Sevilla, Spain; imaza@us.es

**Keywords:** unmanned aerial vehicles, landing on mobile platforms, autonomous landing

## Abstract

This article presents a precise landing system that allows rotary-wing UAVs to approach and land safely on moving platforms, without using GNSS at any stage of the landing maneuver, and with a centimeter level accuracy and high level of robustness. This system implements a novel concept where the relative position and velocity between the aerial vehicle and the landing platform are calculated from the angles of a cable that physically connects the UAV and the landing platform. The use of a cable also incorporates a number of extra benefits, such as increasing the precision in the control of the UAV altitude. It also facilitates centering the UAV right on top of the expected landing position, and increases the stability of the UAV just after contacting the landing platform. The system was implemented in an unmanned helicopter and many tests were carried out under different conditions for measuring the accuracy and the robustness of the proposed solution. Results show that the developed system allowed landing with centimeter accuracy by using only local sensors and that the helicopter could follow the landing platform in multiple trajectories at different velocities.

## 1. Introduction and Related Work

In the next decade, it is expected that civil applications of Unmanned Aerial Vehicles (UAVs) will increase exponentially up to a market of 11,000 million euros in 2035 only in Europe [1]. Moreover, and due to the intrinsic low risk of maritime operations with UAVs, an important increase in the use of UAVs from ships is foreseen for different applications: environmental monitoring, fishing support, surveillance, etc. In operations from ships and boats, the landing maneuver is the phase of the operation that involves a higher risk and where a higher level of precision in the position and velocity estimation, along with a high level of robustness in the operation, is required. Although landing of rotary-wing UAVs has raised the attention of multiple researchers during the last 15 years [2], it has not yet been completely solved in a robust and reliable manner. In fact, one of the three challenges at the Mohamed Bin Zayed International Robotics Challenge (MBZIRC) Competition in 2017 was the landing of an UAV on a moving platform [3].

One of the first research works to tackle the autonomous landing problem on mobile platforms was by Saripalli et al. [4] in 2003, who developed a real-time, vision-based landing algorithm. Later, Saripalli and Sukhatme [5] and Merz et al. [6] presented vision-based algorithms that can estimate the helicopter position from images of a specially designed landing pad with the necessary level of precision and accuracy for the landing maneuver. Most data were gathered from manual flights and simulations. More recently, Falanga et al. [7] and Polvara et al. [8] also applied vision-based landing techniques increasing the autonomy level by including real-time trajectory generation. In addition, it is possible to find recent works that study the problem of landing in an oscillating platform by using visual sensors. For instance, the authors of [8] used a fiducial marker to obtain the pose of the platform and implemented an External Kalman Filter (EKF) to estimate the ship position. Simulations provide very accurate results by using only the odometry and the inertial measurements for the estimation. However, measurements from these sensors may not always be available and they can suffer physical interferences and limitations in their fields of view. Additionally, these approaches generally only work under good light or visibility conditions so their performance has a strong dependency with the weather conditions, seriously limiting their applicability under a wide range of realistic scenarios for the landing operation. Moreover, some of these vision-based techniques assume the use of the Global Navigation Satellite System (GNSS) in their navigation systems, since the vision system is only used as an input that increases the precision of the relative estimation of position and velocity with respect to the landing platform. Therefore, the robustness of these solutions is compromised due to the known problems of current GNSS systems in cluttered environments, such as the deck of the ships.

The use of a cable or tether that physically connects the UAV and the moving platform is not new, although as explained in the following, most existing works are theoretical and only with simulation results. Some works are focused on the study of the control and stabilization problems of tethered rotorcrafts (e.g., [9]). In addition, in [10], a study can be found on the longitudinal stability of a hovering tethered rotorcraft. The first use of a tether for helping in the landing phase of an UAV can be found in [11] where a controller is developed to use the tether tension to couple the translation of the helicopter to the rotation. In [12], the authors described the design of a flight control system architecture for a tethered quad-rotor aircraft where the tether is also used for transmitting power to the UAV. Results from simulated waypoint navigation and hovering of the tethered vehicle suggest that the designed system is fit for use in an automated landing missions. In [13], the authors took advantage of the tensile force acting along the taut cable and solved the nonlinear control of the tethered UAV by using a cascade control scheme based on thrust vectoring and using a novel “Reference Governor scheme”. The work presented in [14] is also focused on the control design problem of a tethered drone and uses the tether as a position sensor. Simulations results show that a full autonomous flight could be achieved indoors by using its approach. However, in all these works, the tether is only used for control and stability purposes and all results and conclusions are obtained from simulations.

There are very few experimental results regarding the use of a tether with an UAV for landing purposes. The first experimental results can be found in [15], where a tethered helicopter lands autonomously over a static landing platform in an outdoor scenario. This work is mainly focused in the control strategy and the advantages that a tether can provide to the stability of an UAV. In [16], a power-feeding tethered micro UAV is used and a position estimation method based on observing the slack tether is proposed. Some indoor experiments are carried out to prove the feasibility of this method. The authors of [17] proposed localizing an UAV in indoor environments by using only a quasi-taut tether. The tether’s sensory feedback is fed into a catenary-based mechanics model to localize the UAV in an indoor global frame defined by the tether reel center. They tested their localization method on a physical robot (Fotokite Pro). Although it is possible to find real experimental data in [16,17], landing tests in these works are performed over static platforms and in indoor scenarios where the weather conditions do not affect the navigation capabilities.

The system developed in this work was designed for landing a rotary-wing UAV (RUAV) on a mobile platform with a high level of accuracy and robustness, and without using GNSS. The design was inspired by the Recovery Assist, Secure and Transverse (RAST) system used by manned helicopters to improve the stability using a tether during the landing operation [18]. This study was a large extension of the preliminary results presented in [19,20,21], where the main contributions are: the use of the tether to estimate the relative position and velocity between the UAV and the mobile platform without using GNSS; the design of a robust guidance approach to perform landings in a safe and robust manner; and the validation of the designed algorithms in a large number of flying tests, including landings with speeds of the mobile platform up to 40 km/h. It is important to point out that the calculation of an accurate estimation of the relative position and velocity of the UAV with respect to the landing platform for this navigation solution is obtained by using an altimeter, the inertial data from an on-board Inertial Measurement Unit (IMU) and the tether orientation. This relative information is completely independent from the GNSS and represents an alternative, low cost and reliable positioning system for tethered helicopter UAVs or multicopters. Therefore, the main contributions of this paper are twofold: It presents a robust estimation method based on a novel tether system designed, manufactured and integrated into a rotary wing UAV that provides relative measurements at 100 Hz with centimeter accuracy, and, to the best of our knowledge, it includes the first field experiments with a tethered unmanned helicopter landing on a mobile platform by using the tether as its only positioning source.

The paper is organized as follows. The developed GNSS-free landing system is described in Section 2. Section 3 details the rotary wing UAV and the equipment used during the real tests, whereas Section 4 presents the tests that were carried out to validate the system developed along with the experimental results. Section 5 closes the paper with the conclusions and future developments.

## 2. GNSS-Free Landing System Description

A typical mission of a RUAV is split in several phases. The helicopter takes-off from its base, it flies to an area for mission execution usually using waypoint navigation, and once the task is completed the RUAV starts the approach to the landing area. Finally, once the helicopter is over the landing location, it starts the descent until it lands. The majority of the navigation strategies employed in autopilots are based on fusing the GNSS information with the navigation solution calculated using the accelerometers and gyros of the inertial measurement unit. In fact, this is the most common strategy for taking-off, and the waypoint navigation phases, where the positioning in absolute coordinates provides enough accuracy for performing the different maneuvers. However, if the landing phase has to be performed on moving platforms, a more robust strategy is required based on more accurate sensors. Moreover, the use of a GNSS-free landing system increases the robustness of the system by providing more sources of positioning, especially in non-GNSS friendly environments.

**Figure 1:**
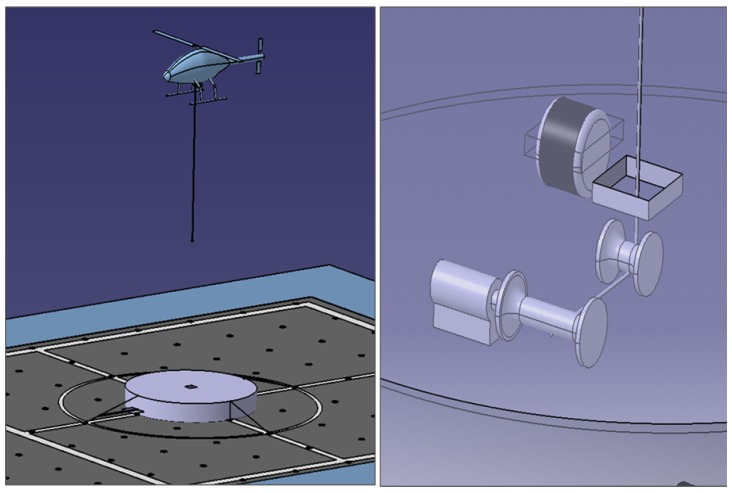
Tether deployment during the rope preparation and detail of the device on-board the landing platform for tether control.

Regarding the different phases of a mission mentioned above, in this work, it is assumed that the helicopter has reached the landing area by using GNSS based navigation and that the rope preparation phase has been completed: the tether has been deployed from the helicopter and locked into the device installed in the landing platform for controlling the tether tension and velocity. This phase is shown in Figure 1.

The work and the experiments presented in this article focused on the landing maneuver of the RUAV. The different steps that compound this phase are summarized below:

- **Initial condition:** The rotary wing UAV is flying autonomously based on GNSS navigation with the tether already attached to the moving platform. The tension applied in the tether is low, so the tether can freely slide.
- **Step 1:** Once the UAV is over the platform, the altimeter is activated and the autopilot maintains the relative altitude to the platform.
- **Step 2:** The ground controller applies a predefined value of tension to the tether to keep the tether straight. This tension is constant during the whole operation.
- **Step 3:** At this status, the tether is tense and the helicopter is maintaining the relative altitude to the platform by using the altimeter. At this moment, it is possible to switch the navigation strategy from GNSS-based to the tether navigation system.
- **Step 4:** The helicopter follows the movements of the landing platform maintaining the relative horizontal position and altitude commanded by the guidance system.
- **Step 5:** The helicopter moves in the horizontal plane towards the origin of the landing platform (point where the tether is attached on ground). In this position, the rope keeps vertical. Once the UAV reaches this point, it is possible to start the descent maintaining a constant vertical velocity.
- **Step 6:** Landing procedure is finished when the rotary wing UAV is standing on the platform and the rotor stops.

### GNSS-Free Navigation Algorithm

One of the main contributions of this work is the relative navigation strategy by using a tether for the landing phase of the rotary-wing UAV. To obtain useful information from the tether, a specific device was developed. It consists of two-axis coupled cardan joints that allow estimating the angles between the tether and the helicopter frame in terms of the two successive rotations and a load sensor to measure the tension level of the tether.

**Figure 2:**
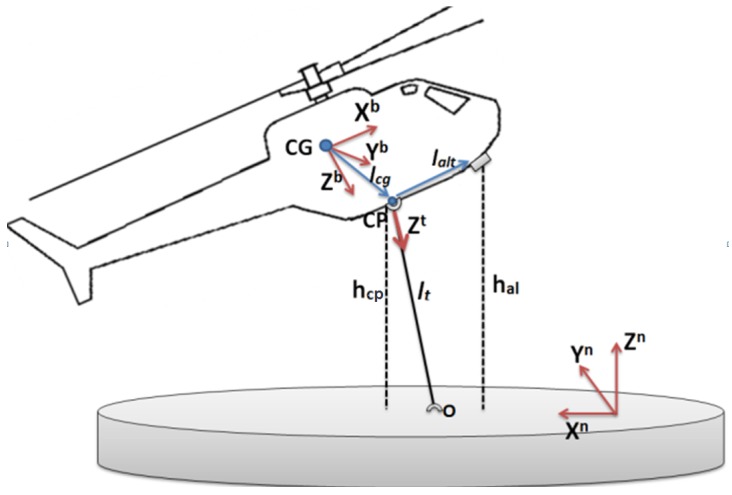
Landing scenario frames and elements that play a relevant role in the landing procedure presented in this paper. The center of gravity of the RUAV is designed as CG, the Contact Point (CP) is the location where the tether system is installed in the fuselage of the UAV, $h_{al}$ is the altitude above ground level (AGL) measured by the altimeter, $h_{cp}$ is the AGL in the CP, $l_{t}$ is the longitude to the landing point O, $l_{cg}$ is the lever arm between the CG and the CP and $l_{alt}$ is the lever-arm between the CP and the altimeter. For the sake of clarity, Figure 3 shows a model of this device with the tether and body coordinate systems represented over it.

Three different reference frames were considered in this study (see Figure 2):

- Body frame (*B*): The body frame is a non-inertial coordinate system associated with the vehicle with the origin at its center of gravity. The *x*-axis points in the forward direction, the *z*-axis down through the vehicle and the *y*-axis completes the right-hand coordinate system. This frame is denoted by the superscript b.
- Local Navigation Tangent Plane frame (*N*): This is an inertial coordinate system determined by fitting a tangent plane to the geodetic reference ellipsoid at a fixed point. This point is taken as the origin of the coordinate system. The *x*-axis points to the true north, the *y*-axis points to the west and the *z*-axis points up. This frame is denoted by the superscript n.
- The tether frame (*T*): It is a non-inertial coordinate system associated with a cardan joint mechanism. It has its origin in the point where the tether is connected to the helicopter. The *x*- and *y*-axes rotate with respect to the fuselage of the helicopter and the *z*-axis is always pointing towards the landing point. This frame is denoted with superscript t.

Figure 2 shows a scheme with the different elements that play a role in the landing procedure presented along this work.

**Figure 4:**
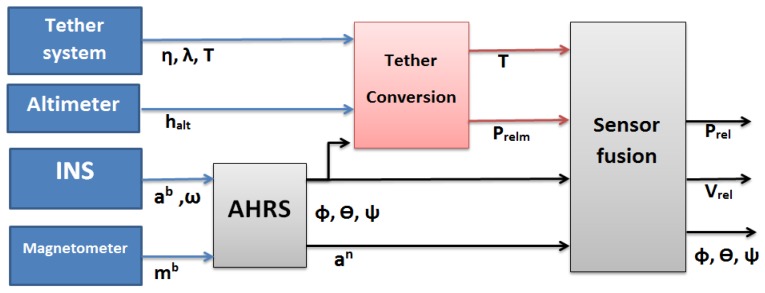
Architecture of the relative estimation system.

The precision obtained by usual algorithms based on fusing a GNSS sensor with an Inertial Navigation System (INS) is not enough to perform a safe approach and landing, especially in moving platforms. Therefore, a technique to estimate the position in real time with high accuracy is needed in order to successfully accomplish the autonomous landing safely. In our study, a relative estimator was developed and implemented in an autonomous helicopter in order to be used during the landing phase on static or mobile platforms. Figure 4 shows the architecture of the relative estimation module. As can be seen, the inputs to this module are the data provided by the tether system (angles η and ρ, and tension T), the altitude of the altimeter ($h_{alt}$), the accelerations and angular velocities of the INS ($a^{b}$ and ω) and the magnetic field measurements of the magnetometer ($m^{b}$). This scheme is composed by:

- Attitude and Heading Reference System (AHRS) block is the module in charge of calculating the attitude of the UAV (roll ϕ, pitch θ and yaw ψ). The attitude is defined as the inclination of its body-axes reference frame to the navigation reference frame. In addition, in this block, the accelerations are rotated to the navigation axes($a^{n}$).
- Tether Conversion block performs all the geometric and rotation operations needed to translate the tether information to a relative position vector ($p_{relm}$).
- Sensor Fusion block fuses all the information obtained from the AHRS and the Tether conversion block and estimates the relative state vector that is used by the controller of the RUAV.

In our work, a crucial requirement for the estimation module is to obtain a precise tracking of the relative position and velocity between the helicopter and the landing platform. Most of the relative kinematics works are based on the fact that both vehicles have an external positioning source (generally GNSS) and they can share their own information through a communication link. This is very common for example in leader–slave architectures for formation flights [22] where the system model uses information of the state vector received from the other vehicles and the relative position measurements are obtained by using a Differential GPS architecture. However, in this work, we did not rely on communication links or external very accurate positioning systems. In this way, as the true behavior of the vehicles was not known, the control input of the relative state vector was modeled as a random process with certain properties.

To build the model for the relative estimation in the approach maneuver, we had to take into account that the filter does not have any information about the dynamic of the landing platform. In this case, we chose to use a stochastic dynamic model of the relative vector between the vehicles, where a random variable represents an unknown time-varying quantity. In particular, our system model is a modified version of a Singer acceleration model. The Singer acceleration model [23] is a popular model [24,25] for target maneuvers that characterizes the unknown target acceleration as a time-correlated stochastic process. It is an a priori model since it does not use online information about the target maneuver, although it can be made adaptive through an adaptation of its parameters. In this case, the acceleration is modeled as a zero-mean first-order stationary Markov process with an autocorrelation function [26]:

(1) $$R_{a}\left(\tau \right)=E\left[a\left(t+\tau \right)a\left(t\right)\right]=\sigma^{2}e^{-a|\tau |},$$

where $\sigma^{2}$ is the variance process noise and α is the reciprocal of a maneuver time constant τ that depends on how long the maneuver lasts, for instance, in a slow turn of an aircraft τ, ∼60 s, and, in an evasive maneuver τ, ∼10–20 s [23]. In a Markov process, its value at a given time depends on values at other times only through its nearest neighbors. To provide values for these parameters, some typical simplifying assumptions for the ship model were taken [27,28]: the landing platform follows a straight trajectory with (nearly) constant velocity. Regarding the helicopter, it was assumed that the autopilot is capable of following the ship in a soft way during the maneuver (this last assumption was proved by the tests that are presented in Section 4). One of the shortcomings of the Singer model is that the acceleration has a zero mean at any moment [26]. However, we could use information from the inertial sensors on-board the RUAV, so some modifications can be done in the model in order to overcome this limitation. In the landing scenario, as the ship was assumed to have a slow dynamic, most of the changes in the relative velocity between both vehicles are due to the accelerations of the helicopter. These accelerations are not zero and can be measured by the accelerometers on-board. Hence, the Singer model can be modified to have a non-zero mean of the acceleration. This approach it is potentially more effective than the Singer model because it includes in the model most of the dynamics of the relative vector that is associated with the helicopter accelerations. In this way, the acceleration model satisfies

(2) $$a\left(t\right)=\bar{a}\left(t\right)+\tilde{a}\left(t\right),$$

where $\bar{a}$ is the mean acceleration of the helicopter in the navigation frame, which was assumed to be constant during the sampling period. On the other hand, $\tilde{a}\left(t\right)$ is the zero-mean Markov process of the Singer model, with the autocorrelation function shown in Equation (1), and it satisfies

(3) $$\dot{\tilde{a}}\left(t\right)=-\alpha \tilde{a}\left(t\right)+w\left(t\right).$$

In Equation (3), *w*(*t*) is modeled as a zero-mean white noise. If $\tilde{a}\left(t\right)$ is expressed in Equation (2) and plugged into Equation (3), it is possible to obtain

(4) $$\dot{\tilde{a}}\left(t\right)=-\alpha a\left(t\right)+\alpha \bar{a}\left(t\right)+w\left(t\right).$$

If we note from Equation (2) that $\dot{\tilde{a}}\left(t\right)=\frac{\partial }{\partial t}\left(a\left(t\right)-\bar{a}\left(t\right)\right)$ and it was assumed that the acceleration of the helicopter $\bar{a}\left(t\right)$ is constant over a sampling interval $T_{s}$, we obtain

(5) $$\dot{a}\left(t\right)=-\alpha a\left(t\right)+\alpha \tilde{a}\left(t\right)+w\left(t\right).$$

Through Equation (5), it is possible to write the complete stochastic differential equation as:

(6) $$\dot{x}\left(t\right)=\left(\begin{array}{ccc} 0 & 1 & 0\\ 0 & 0 & 1\\ 0 & 0 & -\alpha \end{array}\right)x\left(t\right)+\left(\begin{array}{c} 0\\ 0\\ \alpha \end{array}\right)\tilde{a}\left(t\right)+\left(\begin{array}{c} 0\\ 0\\ 1 \end{array}\right)w\left(t\right).$$

In Equation (6), x(t) is the relative state vector [pr(t),vr(t),ar(t)] where pr(t), vr(t) and ar(t) are the relative position, velocity and acceleration, respectively. By applying a standard discretization step in Equation (6), it is possible to obtain the discrete state equation

(7) $$x_{k+1}=F_{\alpha }x_{k}+U_{\alpha }{\tilde{a}}_{k}+w_{k},$$

where $F_{\alpha }$ is the state transition matrix, $U_{\alpha }$ is the discrete time input matrix and $w_{k}$ is a white noise sequence with covariance matrix $Q_{k}$. The values of these matrices are given by Equations (8)–(10).

(8) $$F_{\alpha }=\left(\begin{array}{ccc} 1 & T_{s} & \frac{1}{\alpha^{2}}\left(\alpha T_{s}-1+e^{-\alpha T_{s}}\right)\\ 0 & 1 & \frac{1}{\alpha (1-e^{-\alpha T_{s}})}\\ 0 & 0 & e^{-\alpha T_{s}} \end{array}\right),$$

(9) $$U_{\alpha }=\left(\begin{array}{c} \frac{T_{s}^{2}}{2}-\frac{\alpha T_{s}-1+e^{-\alpha T_{s}}}{\alpha^{2}}\\ T_{s}-\frac{1-e^{-\alpha T_{s}}}{\alpha }\\ 1-e^{-\alpha T_{s}} \end{array}\right)and$$

(10) $$Q_{k}=E\left(w_{k}w_{k}^{T}\right)=2\alpha \sigma_{\alpha }^{2}\left(\begin{array}{ccc} q_{11} & q_{12} & q_{13}\\ q_{12} & q_{22} & q_{23}\\ q_{13} & q_{23} & q_{33} \end{array}\right),$$

where the terms of the process noise covariance matrix (a similar methodology can be found in [29,30], where a velocity model is derived) are given by

(11) $$\begin{array}{c} q_{11}=\frac{1}{2\sigma^{5}}\left(1-e^{-2\alpha T_{s}}+2\alpha T_{s}+\frac{2}{3}\alpha^{3}T_{s}^{3}-2\alpha^{2}T_{s}^{2}-4\alpha T_{s}e^{-\alpha T_{s}}\right)\\ q_{12}=\frac{1}{2\sigma^{4}}\left(e^{-2\alpha T_{s}}+1+2e^{-\alpha T_{s}}+2\alpha T_{s}e^{-\alpha T_{s}}-2\alpha T_{s}-\alpha^{2}T_{s}^{2}\right)\\ q_{13}=\frac{1}{2\sigma^{3}}\left(1-e^{-2\alpha T_{s}}-2\alpha T_{s}e^{-\alpha T_{s}}\right)\\ q_{22}=\frac{1}{2\sigma^{3}}\left(4e^{-\alpha T_{s}}-3-e^{-2\alpha T_{s}}+2\alpha T_{s}\right)\\ q_{23}=\frac{1}{2\sigma^{2}}\left(e^{-2\alpha T_{s}}+1-2e^{-\alpha T_{s}}\right)\\ q_{33}=\frac{1}{2\sigma }\left(1-e^{-2\alpha T_{s}}\right). \end{array}$$

In Equation (10), $\sigma_{\alpha }$ is a conditional density modeled as a Rayleigh distribution variance of the acceleration for $a_{k+1}$ and its equation is:

(12) $$\sigma_{\alpha }^{2}=\left\{\begin{array}{cc} \frac{4-\pi }{\pi }{\left(a_{max}-\hat{a}\left(t\right)\right)}^{2}, & \hat{a}\left(t\right)>0\\ \frac{4-\pi }{\pi }{\left(-a_{min}-\hat{a}\left(t\right)\right)}^{2}, & \hat{a}\left(t\right)<0 \end{array}\right.,$$

where $\hat{x}$ is the current predicted acceleration and $a_{max}$ and $a_{min}$ are design parameters that correspond to the maximum and minimum accelerations, respectively. In this equation, if the absolute values of the design parameters are small, the accuracy of the estimation will tend to be high; however, the filter will have a slow response in cases where the changes of the relative motion are very aggressive. On the other hand, if the maximum and minimum values are larger, the model allows a quick response to the dynamics changes but the tracking accuracy becomes lower. In this work, the helicopter can follow the mobile platform in a soft manner, thus it was preferred to model these acceleration parameters using a low profile in order to obtain more accurate estimations. By generalizing the one-dimensional Equation (6), the equations of the model for three dimensions are given by

(13) $$x_{k+1}=\left(\begin{array}{ccc} F_{\alpha } & 0_{3x3} & 0_{3x3}\\ 0_{3x3} & F_{\alpha } & 0_{3x3}\\ 0_{3x3} & 0_{3x3} & F_{\alpha } \end{array}\right)x_{k}+\left(\begin{array}{ccc} U_{\alpha } & 0_{3x1} & 0_{3x1}\\ 0_{3x1} & U_{\alpha } & 0_{3x1}\\ 0_{3x1} & 0_{3x1} & U_{\alpha } \end{array}\right){\tilde{a}}_{k}+w_{k}.$$

Once the discrete time dynamic equations have been modeled, the measurement equation of the discrete-time system is presented as:

(14) $$z_{k}=H_{k}x_{k}+v_{k}$$

where $z_{k}$ is the measurement vector that contains the relative positions between the rotary-wing UAV and the landing point calculated using the tether system, $H_{k}$ is the measurement matrix and $v_{k}$ is the noise in the measurements. The measurement matrix is given by

(15) $$H_{k}=\left(\begin{array}{ccccccccc} 1 & 0 & 0 & 0 & 0 & 0 & 0 & 0 & 0\\ 0 & 0 & 0 & 1 & 0 & 0 & 0 & 0 & 0\\ 0 & 0 & 0 & 0 & 0 & 0 & 1 & 0 & 0 \end{array}\right).$$

Figure 2 presents the different elements that are used by the Tether Calculation Block shown in Figure 4 for the computation of the vector z. The first step for obtaining the relative positioning measurements consists on calculating the altitude to the landing point from the contact point. Because the altimeter is not installed in the same place, to have as much accuracy as possible, it is necessary to correct the lever arm according to the equations

(16) $$R_{b}^{n}={\left(R_{n}^{b}\right)}^{t}=\left(\begin{array}{ccc} c\theta c\psi & -c\phi s\psi +s\phi s\theta c\psi & s\phi s\psi +c\phi s\theta c\psi \\ c\theta s\psi & c\phi c\psi +s\phi s\theta s\psi & -s\phi c\psi +c\phi s\theta s\psi \\ -s\theta & s\phi c\theta & c\phi c\theta \end{array}\right)and$$

(17) $$h_{CP}=h_{al}-R_{b}^{n}\left(3,:\right)l_{alt}=h_{al}-s\theta l_{x}+s\phi c\theta l_{y}+c\phi c\theta l_{z},$$

where $R_{b}^{n}$ is the rotation matrix from the body to the navigation frame calculated in the AHRS block and “*c*” and “*s*” denotes cosine and sine, respectively. The cardan joint is rigidly attached to the helicopter and perfectly aligned with the $X^{b}$ and $Y^{b}$ body axes of the vehicle. This device rotates η and ρ angles with respect to the helicopter fuselage around its $X^{b}$ and $Y^{b}$ axes, respectively, thus the rotation matrix from the tether to the body frame is

(18) $$R_{t}^{b}=\left(\begin{array}{ccc} c\rho & 0 & -s\rho \\ -s\eta s\rho & c\eta & -s\eta c\rho \\ c\eta s\rho & s\eta & c\eta c\rho \end{array}\right).$$

Once the altitude h_CP_ has been calculated, it is necessary to compute the relative position in navigation axes from the CP to the landing point as

(19) $$P_{CP}^{n}=R_{b}^{n}R_{t}^{b}P_{CP}^{t},$$

where $P_{CP}^{n}$ and $P_{CP}^{t}$ are the positions vectors of the contact point in the navigation and the tether frame, respectively. In the tether frame, the horizontal XY coordinates of the landing point are 0 so the position vector is expressed as [0, 0, *z^t^* ] and Equation (19) can be written as:

(20) $$\left(\begin{array}{c} x_{CP}^{n}\\ y_{cp}^{n}\\ h_{CP} \end{array}\right)=R_{t}^{n}\left(\begin{array}{c} 0\\ 0\\ z^{t} \end{array}\right).$$

By using Equation (20), it is possible to obtain the relative coordinates of the contact point in the navigation frame as

(21) $$z^{t}=\frac{h_{CP}}{R_{t}^{n}\left(3,3\right)},$$

(22) $$x_{CP}^{n}=R_{t}^{n}\left(1,3\right)z^{t}and$$

(23) $$y_{CP}^{n}=R_{t}^{n}\left(2,3\right)z^{t}.$$

The last step is to translate this relative position to the center of mass (CG) of the vehicle by applying another lever arm correction:

(24) $$P^{n}=P_{CP}^{n}-R_{b}^{n}l_{CG}.$$

Once the relative position vector has been computed from the sensors outputs, it can be used as the measurement vector $z_{k}$ of Equation (14). Because this vector has been calculated through rotations and translations to work in the navigation frame, the components of the measurement noise covariance become correlated. To calculate the terms of the covariance matrix, it is necessary to calculate the error propagation in a multi-input multi-output system. The measurement noise covariance matrix has the form:

(25) $$R_{k}=\left(\begin{array}{ccc} \sigma_{xx}^{2} & \sigma_{xy}^{2} & \sigma_{xz}^{2}\\ \sigma_{xy}^{2} & \sigma_{yy}^{2} & \sigma_{yz}^{2}\\ \sigma_{xz}^{2} & \sigma_{yz}^{2} & \sigma_{zz}^{2} \end{array}\right),$$

where the terms of the diagonal of the covariance matrix can be calculated as:

(26) $$\sigma_{xx}^{2}=\sum_{i}^{}{\left(\frac{\partial f}{\partial s_{i}}\right)}^{2}\sigma_{i}^{2}+\sum \sum_{i\neq j}^{}\left(\frac{\partial f}{\partial s_{i}}\right)\left(\frac{\partial g}{\partial s_{j}}\right)\sigma_{ij},$$

where *f* is the measurement function presented in Equation (24), $\frac{\partial f}{\partial s_{i}}$ denotes the derivate of the function f with respect to the *i*th sensor, $\sigma_{i}^{2}$ represents the variance of the *i*th sensor and $\sigma_{ij}$ is the covariance between the *i*th and *j*th sensors. In this case, the sensors are independent so the covariance $\sigma_{ij}$ disappears and the resulting variance is

(27) $$\sigma_{xx}^{2}=\sum_{i}^{}{\left(\frac{\partial f}{\partial s_{i}}\right)}^{2}\sigma_{i}^{2}.$$

For the correlated terms of the covariance matrix, it is possible to calculate the terms solving the equation

(28) $$\sigma_{xy}=\sum_{i}^{}\left(\frac{\partial f}{\partial s_{i}}\right)\left(\frac{\partial g}{\partial s_{j}}\right)\sigma_{i}^{2}+\sum \sum_{i\neq j}^{}\left(\frac{\partial f}{\partial s_{i}}\right)\left(\frac{\partial g}{\partial s_{j}}\right)\sigma_{ij},$$

where the second term can be eliminated because the sensors are independent. Once Equations (27) and (28) are solved, the covariance matrix become extremely complex and it changes every time step. This model involves a very high computational load because the functions that compound each term of the covariance matrix involve many trigonometric functions. After a simulation stage and a post-processing of real data captured during the preliminary experimental phase, it was obtained that this complexity was not necessary, so a simplification was made and the noise covariance matrix was modeled as

(29) $$R=\left(\begin{array}{ccc} \sigma_{h}^{2} & 0 & 0\\ 0 & \sigma_{h}^{2} & 0\\ 0 & 0 & \sigma_{v}^{2} \end{array}\right),$$

where the values of $\sigma_{h}^{2}$ and $\sigma_{v}^{2}$ are given taking into account the accuracy of the relative measurements calculated during the laboratory tests and the current value of the tether tension (high tension is related to a better accuracy). In fact, using these values showed better performance of the filter than using the covariance matrix calculated with Equations (27) and (28). Hence, the measurement noise covariance matrix chosen for the final tests was Equation (29).

Finally, for obtaining the solution of the estimation problem, a linear Kalman filter [31,32] with Equations (6) and (14) is used. This filter will have as output an accurate estimation of the relative state vector at 100 Hz that will be used as the inputs of the control module of the helicopter autopilot.

## 3. Experimental Setup

**Figure 5:**
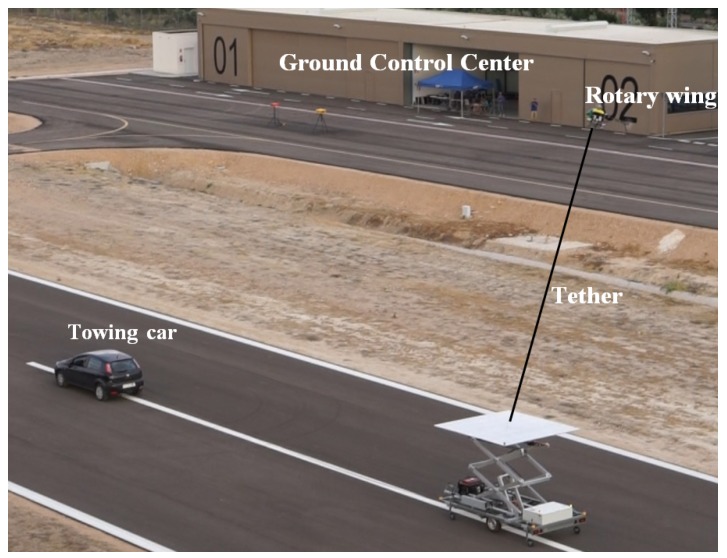
Actors of the complete experimental setup during a landing test.

The experimental setup is significantly complex due to the interaction of many systems that were specifically designed for this research work. In this section, the setup is explained with all the actors implied in the tests. Figure 5 shows the complete setup which is composed by:

- A moving platform mounted on a trolley towed by a vehicle with a tether. The platform moved vertically by means of an elevator frame pushed by an electric engine. This structure also carried a tension controller with a PC unit powered by a generator.
- A car that tows the trolley with a tether.
- A rotary wing UAV: With avionics equipment, and joined to the platform with the tether.
- Tether system: Cardan sensor on-board the UAV and reel system in the platform.
- Ground Control Center, which includes the Ground Control Station for the UAV and the Ground Control Unit of the moving platform, since the platform can be remotely controlled in both its vertical movement and the tether tension.
- Human resources for the complete operation.

The experimental phase had several stages. First, the rotary-wing platform with its avionics equipment was tested until a good autonomous performance was obtained. Afterwards, the tests with the moving platform were split into the following stages:

(a) Autonomous operation using relative coordinates: Validation of relative coordinates navigation, being guided by the tether attached to the platform, which in this stage did not move.
(b) Static landing: Landing of the rotary wing UAV on the platform using relative coordinates. In this phase, the platform was also completely static.
(c) Landing moving horizontally: Landing of the rotary wing UAV on the platform using relative coordinates. The platform movesdin the horizontal frame (towed by a vehicle).
(d) Landing moving vertically: Landing of the rotary wing UAV using relative coordinates on the platform, which in this case moved in the vertical plane (pushed by an engine).
(e) Landing combining all movements: Landing of the rotary wing UAV on the platform, which moved both vertically and horizontally. This landing was guided by the tether using relative coordinates.

These steps were followed sequentially, increasing gradually the level of difficulty to reduce the risks. Tests (a)–(d) can be considered as initial tests for the final Test (e), in which a combination of the previous tests was done.

### 3.1. Airfields

The tests were performed on two different airfields. The preliminary tests were carried out in an airfield located in Utrera (Sevilla, Spain: 37${}^{\circ }$11${}^{\prime }$49.92″ N 5${}^{\circ }$52${}^{\prime }$50.04″ O). It has an unpaved landing strip with a north–south orientation (18–36). The first test exercises were simple subsets of the final tests. The final tests with the platform moving in all axes were performed in ATLAS aerodrome. ATLAS is a test flight center located in Villacarrillo (Jaen, Spain: 38${}^{\circ }$$8^{\prime }16.7382^{\prime \prime }$ N, $3^{\circ }$$10^{\prime }25.8486^{\prime \prime }$ O) specially designed for light and tactical UAS operations.The ATLAS runway is 600 m long and 18 m wide, orientation 07–25, and entirely asphalted.

### 3.2. Rotary-Wing UAV

**Figure 6:**
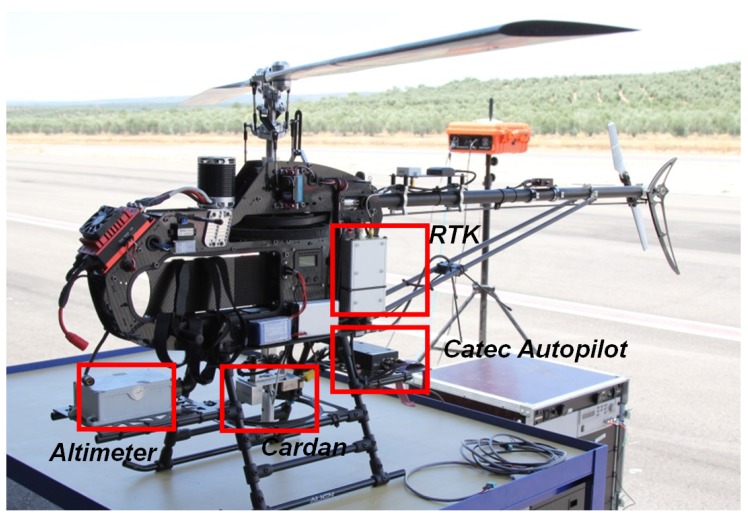
Rotary wing UAV LOGO 800 avionics.

The tests were performed with a rotary wing unmanned helicopter based on the LOGO-800 RC helicopter. This UAV has a very good performance due to low level of vibrations in the airframe, large payload capacity (take-off weight of 15 kg approximately), and high power to weight ratio, which implies a very good dynamic response. The helicopter has a rotor diameter of 1.8 m and a main rotor speed of approximately 1400 rpm powered by a 4.8 kW electrical engine. These aspects make this UAV very suitable for the tests, which require powerful and agile maneuvers. However, it is important to notice that, for safety reasons, this helicopter must not be flown with wind velocities over 25 km/h. To land in a more demanding scenario, it would be necessary to fly with a different aerial platform. The generic equipment consists of the autopilot developed for this work that uses a ten degrees of freedom Inertial Sensor (model ADIS16407 by Analog Devices, Norwood, MA, USA) that measures angular velocities, accelerations and the magnetic field. In addition to these systems, some additional sensors have been installed in the rotary-wing UAV in order to be able to land safely by using the relative positioning navigation scheme presented previously (see Figure 6).

**Figure 7:**
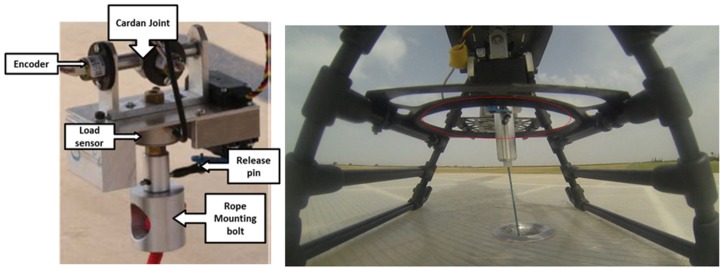
Cardan sensor integrated in LOGO 800 with the tether attached.

- A ROKE MRII laser altimeter was equipped in order to have a centimeter level of accuracy in the relative altitude between the RUAV and the landing platform.
- A centimeter precision Real Time Kinematics (RTK) GNSS system was also integrated into the RUAV in order to have a reference for the positioning and velocity solution obtained by using the relative configuration. These measurements were not used for estimation purposes, only as ground truth for comparison of the results obtained and benchmarking of the developed solution. The selected RTK board was the OEM628 model by Novatel. The accuracy calculated as the Root Mean Square (RMS) of this sensor in the horizontal plane is 1 cm when the RTK is working and its solution is fixed.
- A specific cardan joint device was developed for this work (see Figure 7). This device is similar to those used for slung loads transportation by cooperative helicopters in [33]. The device consists of two-axis coupled cardan joints equipped with magnetic encoders attached to each axis. This system allows estimating the angles between the tether and the helicopter frame in terms of the two successive rotations of the cardan joint. Additionally, the device has a load sensor to measure the tension level of the tether and a tether release system for safety purposes. This safety mechanism produces the separation between the helicopter and the tether and it can be directly activated from the safety pilot radio in the case of emergency. In addition, to avoid dangerous situations with the cable, a protective frame (indicated with red dots in Figure 7) was integrated to assure that the tether did not reach the skids of the helicopter, thus limiting the maximum cardan angle measured and the maximum distance under the estimations are correct. These constraints are used in the guidance block in order to limit the relative references that are provided to the controller. In that manner, the autopilot is able of maintain always the helicopter in the range where the estimations are accurate.

The helicopter was tethered with a Dyneema tether, which can hold a tension up to 80 kg. The tether was attached to the cardan sensor onboard the unmanned helicopter.

As shown in Figure 7, the tether is guided through a small hole on the platform, and collected in a reel, which is controlled remotely.

### 3.3. Landing Platform

The landing pad structure has a dimension of 3 m × 3 m and it is composed of three detachable transparent reinforced panels. An electric engine located in the base moves the spindle and transmits its rotational movement to vertical movement through a scissor pattern structure. This allows the platform to perform movements in the vertical axis with amplitude oscillations up to 4 m and a maximum vertical velocity of 0.5 m/s. The goal was to replicate qualitatively the motion of a ship’s deck under sea conditions below Sea 5 following the Beaufourt scale. Appendix A presents the assumptions taken into account and the limitations regarding the vertical motion capabilities of the landing platform in order to replicate a ship’s deck under different weather conditions.

**Figure 8:**
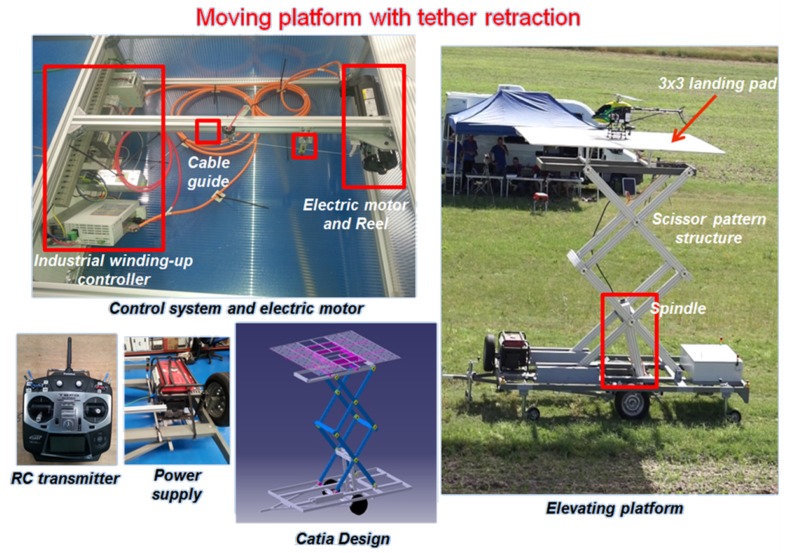
Components of the moving platform.

The control of the platform was commanded via a Radio Control (RC) transmitter, a logic PLC and a communication link. Hence, the vertical movement of the platform can be remotely commanded in real time during the operation. The different components of the moving platform are shown in Figure 8).

## 4. Tests and Experimental Results

As stated in Section 2, the real tests were focused on the landing phase of the mission. Hence, the initial state of the system was the rotary-wing UAV flying autonomously with GNSS and the tether locked in the platform ready to be stretched before the switch to relative coordinates navigation.

**Figure 9:**
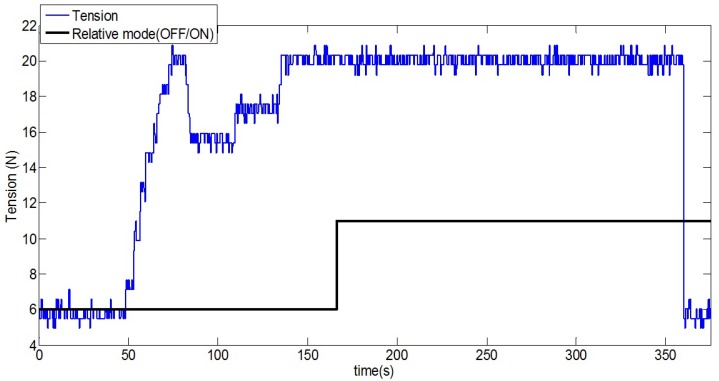
Relative mode is switched on if the tension is bigger than a threshold value during a predefined time interval.

The key of the operation is to have the tether tense when navigating with relative coordinates, so that the tether accurately represents the straight line that connects the helicopter and the origin of the platform, from which the tether is pulled. If the tether is not tense, the cardan angles data will not be representative of the direction vector of the straight line, which connects helicopter and platform, so the relative position estimation would be incorrect. Figure 9 shows the tension and switching signals logged during one test. As can be seen, only when the tension of the tether is at its maximum value does the relative mode turns on.

This section is focused in the description and analysis of the tests that were performed to validate the developed system. The obtained results show that the technique was robust. In fact, the autonomous landing of a rotary-wing UAV guided with a tether on a moving platform was demonstrated with more than 30 successful landings during two different testing campaigns. Some telemetry results are presented in the following to illustrate the performance of the developed system. A video that summarizes the tests performed during the last campaign of flights can be found at https://youtu.be/iJXoHa8EITc.

Tests were carried out in two different months of the year (October and June) and in two different locations allowing to perform the flights under different environmental conditions. This helped to check whether the Guidance, Navigation and Control (GNC) system was robust under different conditions. The range of the wind speed during the tests was from 5 to 20 km/h, so according to the Beaufort scale (see Appendix A), the helicopter flew under wind conditions that reached up to Sea 3 (Gentle Breeze). It is also interesting to point out that the temperature mean in October was 12 ${}^{\circ }$C while in June it was approximately 30 ${}^{\circ }$C. Changes in temperature are important since the density and pressure of the air are key factors for the lift capacity of the helicopter rotor blades. On a warm day, the density is low and the collective pitch for the rotor blades in order to keep hovering position is higher than on a cold day, with higher density of air. Hence, it was also tested whether this architecture was robust under different temperature conditions.

Due to the intrinsic risk of the landing procedure and the characteristics of the small helicopter used, tests with wind velocities over 20 km/h were not carried out. However, in Appendix B, some simulation results under a wider range of wind velocities can be found, showing how the rope system improved the stabilization and safety capabilities of the helicopter when compared with a non-tethered helicopter.

### 4.1. Landing on a Static Platform

**Figure 10:**
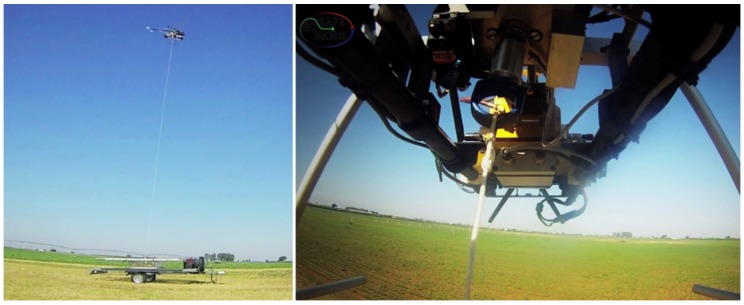
Flight tests with the static platform in Utrera airfield.

In this scenario, the landing platform remains static, and the main goal was to land the helicopter using the relative coordinates obtained with the tether (without using GNSS). These tests were performed at the very beginning of this work in the Utrera airfield and allowed us to obtain sets of telemetry data for improving and tuning of the algorithms before starting the tests campaign in which the landing platform was in movement. All these tests started with the UAV taking off and the tether already attached but with a very low level of tension. Once the helicopter was close to the landing point, the operator commanded to increase progressively the tension of the tether. Once an appropriate value of tension was reached, the helicopter started its landing procedure. Figure 10 shows the RUAV during a landing maneuver.

**Figure 11:**
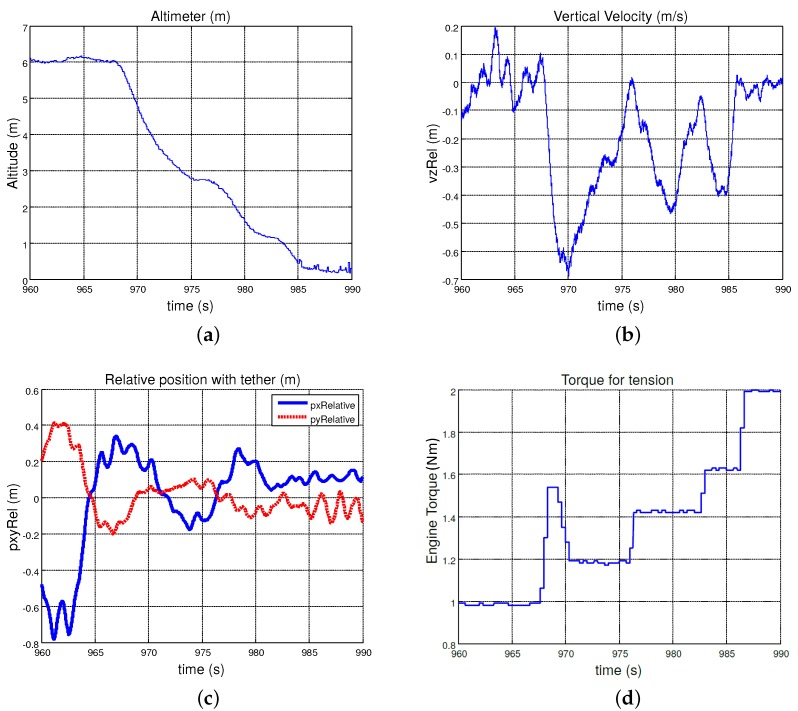
Relation between: altitude (**a**), vertical velocity (**b**) and the horizontal relative position (**c**) with the torque (**d**) applied to the tether in a landing procedure over a static platform by using the developed tether sensor as the positioning system.

Figure 11 shows telemetry data for a landing procedure that used the relative navigation solution provided by the estimator. At the beginning of the landing, when the helicopter was hovering at 6 m AGL, the error in horizontal relative navigation was larger than when the helicopter started to descend. This was due to the “balloon effect” of the helicopter linked with the tether to the platform: while the tether was rolled up, the horizontal displacement of the helicopter became smaller helping the controller to guide the helicopter towards the relative coordinates (0,0) of the platform. This test was performed pulling the tether with the collective saturated, and increasing the tension of the tether. As shown in Figure 11, the torque applied in the electric engine rolled the tether up and eventually defined the tension in the tether; when the tension increased (approximately *t* = 968 s), the helicopter started to descend, and reached a descending velocity of 0.7 m/s. At this moment (*t* = 970 s), the tension applied to the tether was decreased slightly to descend more slowly. At the end of the operation, the helicopter touched down in the origin of the platform, with a very small error (less than 0.2 m) and with very high tension in the tether to keep the helicopter on ground until the engine was shut down.

**Figure 12:**
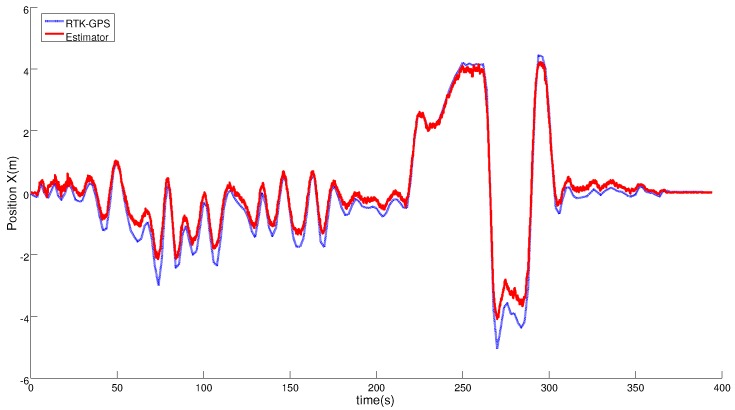
Comparison of the Horizontal Position solution obtained with the estimator (red line) and the RTK-GNSS sensor (blue line) during the complete test.

**Figure 13:**
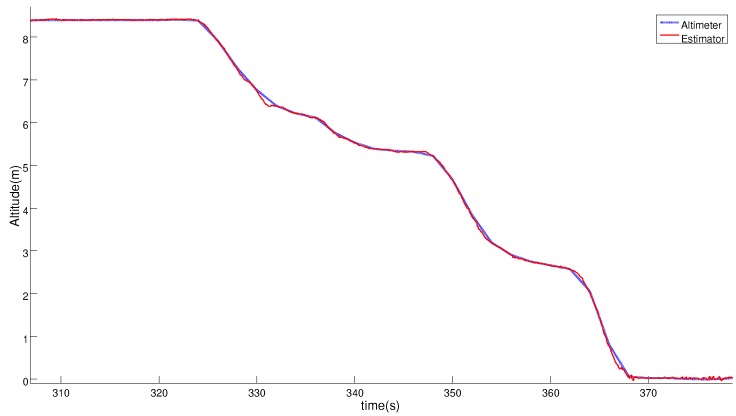
Comparison between the Vertical Position obtained with the estimator (red line) and the altimeter (blue line) in the last phase of the landing test.

Another landing maneuver over the static platform is shown to study the accuracy of the relative estimation solution and to show that it was valid for performing an accurate and safe landing. Figure 12 and Figure 13 compare the position solution of the relative navigator with the measures of the RTK system. These plots show that the estimated relative position has centimeter level accuracy, similar to the RTK system.

Table 1 shows the root mean square error and the standard deviation (STD) of the estimated relative position. As can be seen, the positioning errors were below 20 cm, and hence the accuracy was good enough for landing safely in a platform without using a GNSS sensor.

**Figure 14:**
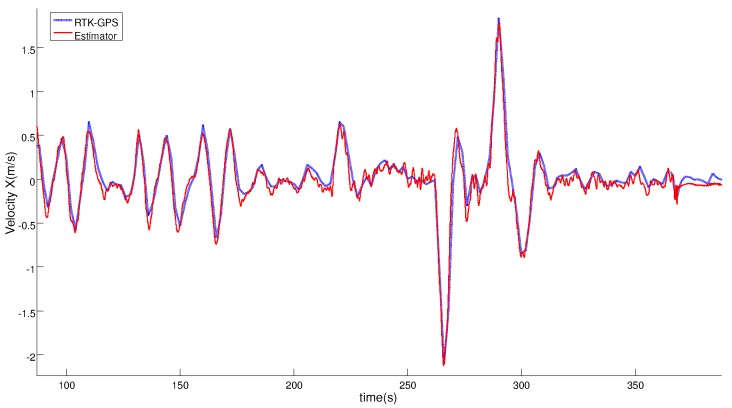
Comparison between the Horizontal velocity solution obtained with the estimator (red line) and the RTK-GNSS sensor (blue line) during the complete test.

**Figure 15:**
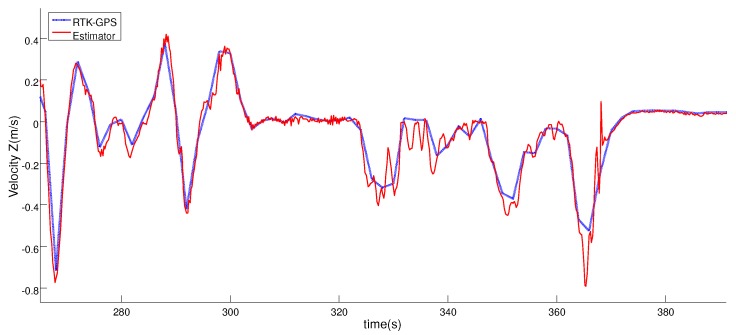
Comparison between the Vertical velocity solution using the estimator (red line) and the velocity solution (blue line) during the complete test.

Figure 14 and Figure 15 compare the relative velocity solution of the relative estimator and the RTK-GPS measurements in the vertical and horizontal planes. As can be seen, the velocity solution obtained by using the tether system was also very accurate. In Table 2, the calculated errors for the relative velocity are shown.

These tests showed that another advantage of the tether is the capability of compensating the ground effect and the lift force of the helicopter when it is landed, allowing the RUAV to remain static over the landing platform until the avionics are switched off.

### 4.2. Landing on a Moving Platform

As mentioned above, during the first experimental phase, one of the objectives was to obtain telemetry data for improving the system performance through a post-processing work in our laboratory. With this feedback, the algorithms were tuned and some modifications in the code were inserted. Once these improvements were performed and implemented, a second campaign of tests in the ATLAS airfield started. Below, some of the tests carried out during this campaign are described. In this campaign, the landing platform was moving with different velocities and following different types of trajectories in the horizontal and vertical planes. In this manner, it was possible to shown that:

- The autopilot can follow the landing platform in a safe and robust manner, allowing the RUAV to reach the references provided by the estimation module.
- The modified Singer model used in the navigation block provides an accurate solution also in scenarios where the landing platform is following trajectories or dynamics that are different to a straight route with constant velocity.

#### 4.2.1. Straight Maneuver

**Figure 16:**
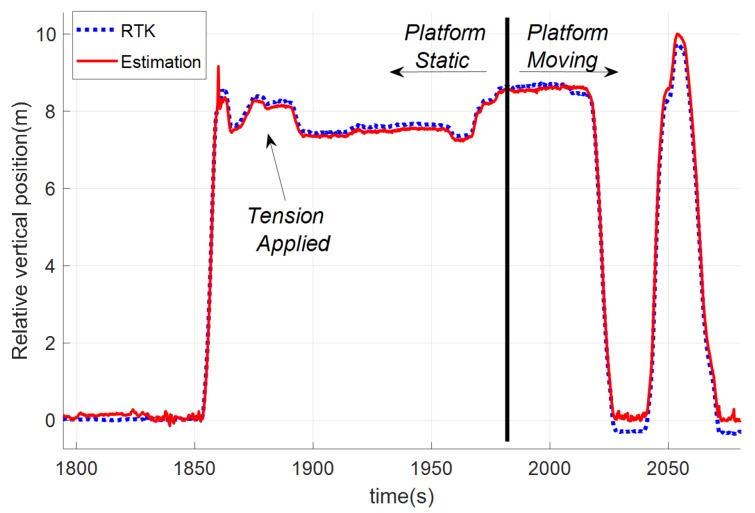
Relative vertical position during the straight movement test and its different phases: static platform, tension applied to the tether, and start of the landing platform motion.

In this test, the landing platform was towed by a car following a straight line. It comprised two different phases: in the first one, the car remained static, the helicopter took off and the tether was tensed. The second phase started with the movement of the car. Figure 16 shows these phases and compares the relative vertical position obtained by using the developed estimator with the relative altitude obtained from the RTK sensors installed in the RUAV and the landing platform. In this test, the UAV landed twice, and the second take-off was done during the movement.

**Figure 17:**
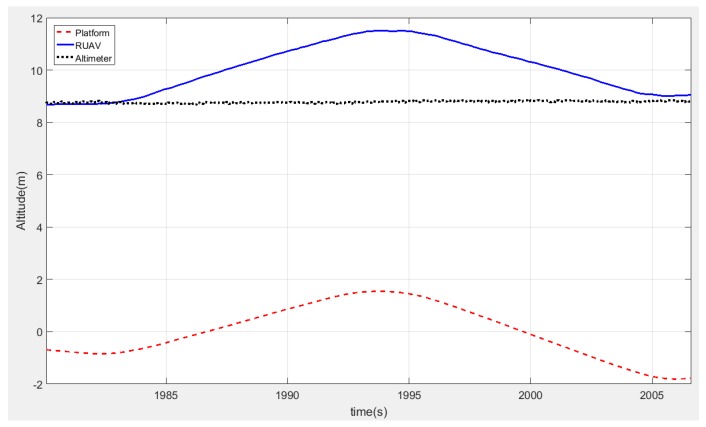
Rotary wing UAV keeps its relative altitude to the moving platform when it oscillates vertically. The altitude of the helicopter in North-West–Up (NWU) coordinates is shown in red, the platform coordinates in NWU coordinates is shown in blue, and the relative altitude between both systems measured by the altimeter is depicted in black.

Here, it is important to notice that, when the platform oscillated vertically, the helicopter maintained the distance with respect to the moving platform. Figure 17 shows the relative altitude provided by the altimeter (black line), the altitude in the local navigation frame of the helicopter (blue line) and the moving platform (red line) during one oscillation of the platform. In this figure, it can be seen how the relative altitude state remained constant at the same time that the landing platform was oscillating in the *z*-axis. It was shown how the estimator allowed flying autonomously at a constant relative altitude in scenarios where the landing platform was moving in the vertical axis.

**Figure 18:**
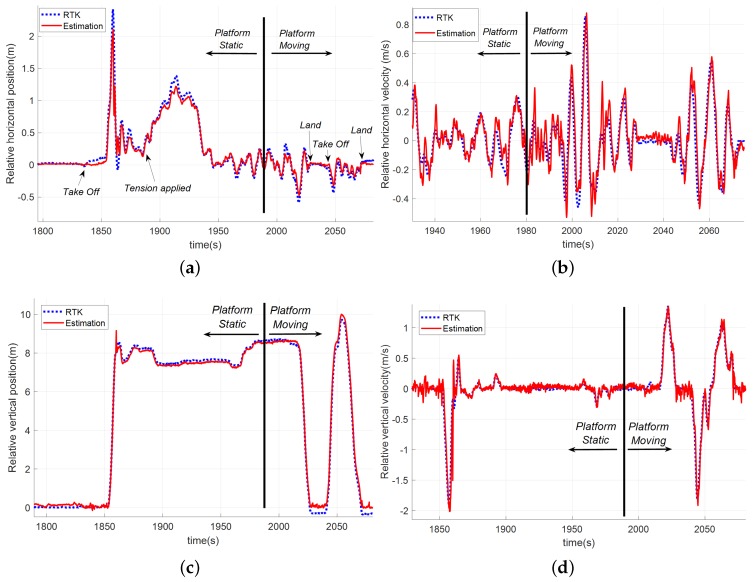
Comparison among the tether based estimation solution (red) and the GNSS-RTK measurements (blue) during a test where the landing platform is moving following a straight trajectory: (**a**) position in the horizontal plane; (**b**) relative velocity in the horizontal plane; (**c**) relative altitude; and (**d**) relative vertical velocity.

To study the estimation accuracy in these tests, Figure 18 shows the comparison between the relative position calculated with the GNSS-RTK system (used again as ground-truth) and the relative position estimation obtained by using the tether.

To calculate the accuracy for the different dynamics, errors were computed bu splitting the tests into the static and moving stages. In that way, it was possible to obtain more precise knowledge about the performance of the filter for the different scenarios.

**Figure 19:**
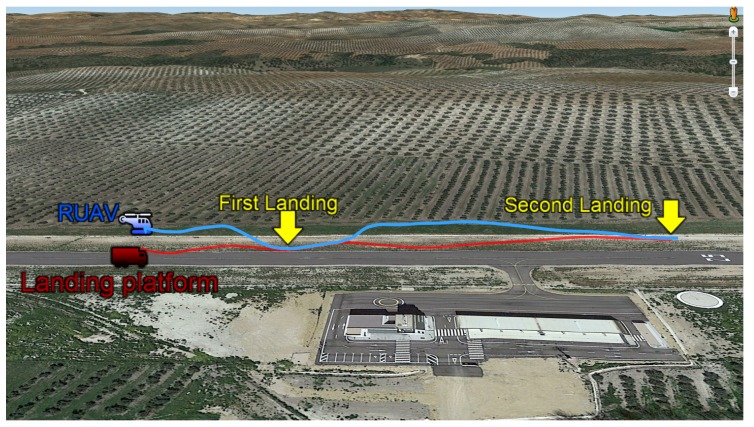
Rotary wing UAV trajectory landing on the moving platform using the tether in the straight trajectory test.

From the results in Table 3, it can be seen that the performance of the filter in the static phase was improved with respect to the first campaign of tests. In this case, the accuracy in both phases was better than 15 cm in position and 0.1 m/s in velocity, allowing the controller to perform the approach and land operations in a precise and safe manner. Figure 19 shows the trajectory of both the helicopter and the moving platform during the maneuver in the phases where the navigation module was using the tether information.

**Figure 20:**
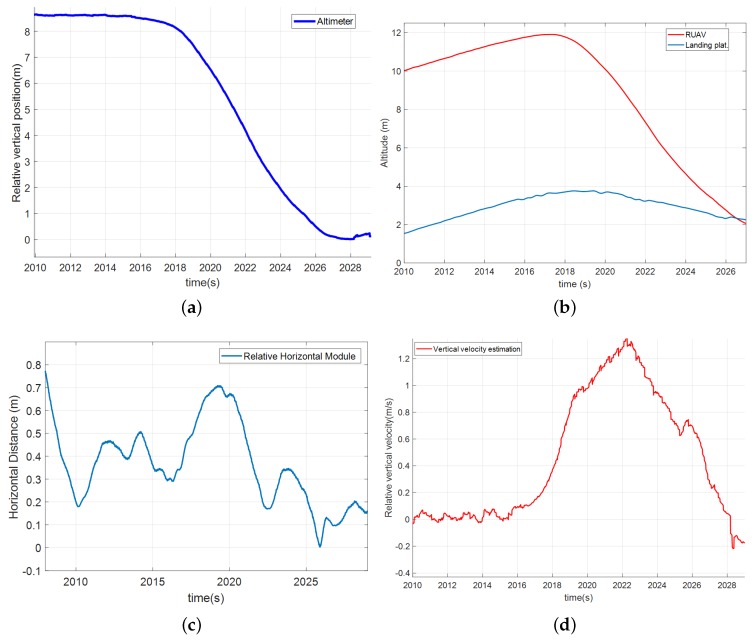
Helicopter descent phase in the straight movement test: (**a**) the altimeter measurements of the relative altitude; (**b**) the AGL of the RUAV and the moving platform; (**c**) the horizontal distance between the helicopter and the landing point; and (**d**) the vertical velocity during the descent.

To analyze the behavior of the autopilot system during the approach maneuver, Figure 20 shows the descent phase of the first landing maneuver. Relative altitude (a) was maintained until approximately *t* = 2017 s, when the descent started and the helicopter got closer to the platform. Figure 20 shows the vertical velocity relative to the moving platform (d), where it is shown that the maximum velocity reached 1.5 m/s as maximum value and this value decreased slowly allowing a smooth landing. In this case, the descent was not performed pulling the RUAV by increasing the tether tension from the electric engine installed in the landing platform, but using the relative altitude information and keeping a constant value of tension. It was shown that descending using this methodology offers a more accurate and soft descent. Apart from that, the relative coordinates calculated with the tether show that the helicopter was kept during the whole maneuver very close to the relative origin (c), thus it remained vertical to the platform.

Another fact that must be taken into account is the ground effect when the helicopter is close to the platform. At that moment, the tension applied to the tether increased very strongly since it had to compensate both the helicopter lift and the ground effect, which pushes the helicopter upwards. This last phase was critical in the operation since the aerodynamic disturbances in the helicopter rotor due to ground interaction made the control much more complex.

**Figure 21:**
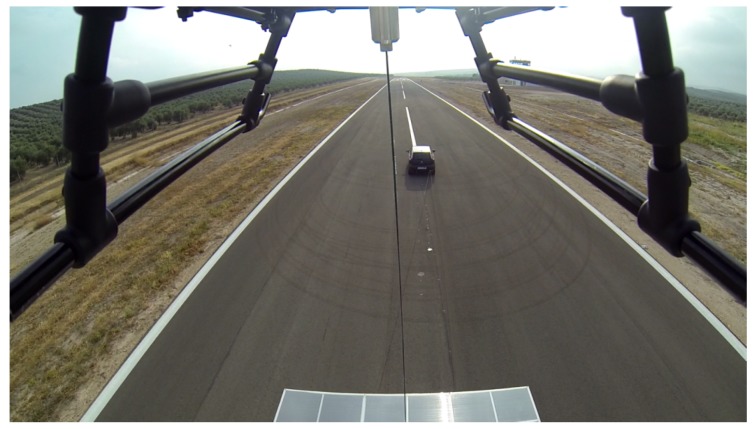
Helicopter goes downs vertical to the platform.

About 20 landings were performed successfully, showing the reliability of the maneuver using relative coordinates with the tether when the landing platform was following a straight trajectory. Figure 21 depicts one image taken from the helicopter some seconds before landing.

#### 4.2.2. Non-Straight Maneuver

**Figure 22:**
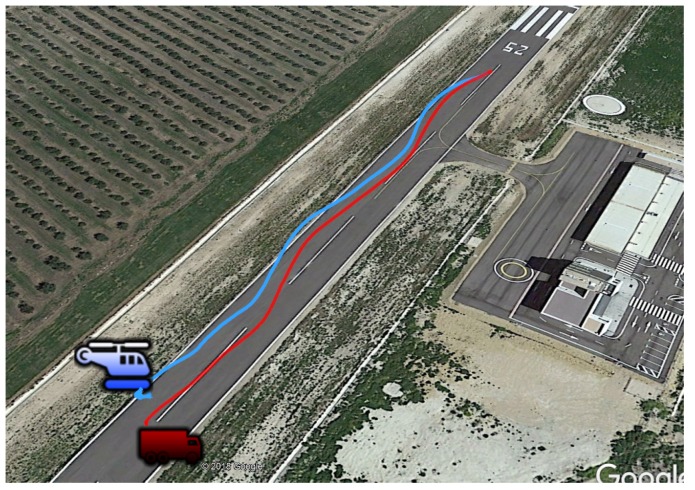
Curvilinear trajectory of the rotary wing UAV for landing on the moving platform using the tether.

In this scenario, the car towed the trolley along curvilinear paths, as can be seen in Figure 22, and the goal was to test the performance of the model developed for the estimation filter in the case that the trajectory of the landing platform was not straight. In this manner, it was possible to confirm that the helicopter was able to land also in vehicles that are performing more complex maneuvers.

**Figure 23:**
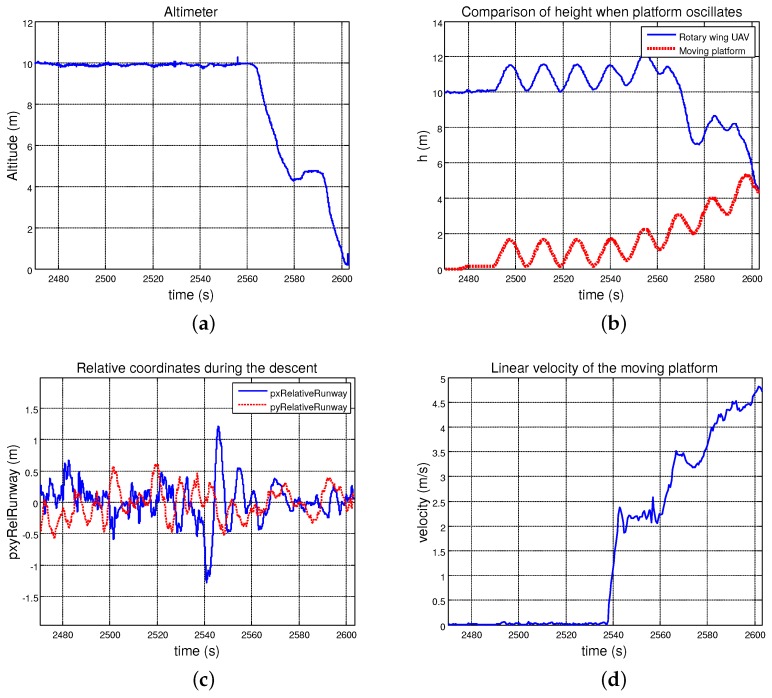
Landing maneuver data regarding curvilinear paths: (**a**) relative altitude measured by the altimeter; (**b**) comparison between the AGL of the RUAV and the moving platform; (**c**) relative distance in the horizontal plane between the helicopter and the landing point; and (**d**) linear velocity of the moving platform during the test.

As can be seen in the following plots, the performance of the autopilot system was quite similar to the one obtained in the previous test. Figure 23a,b, shows how the helicopter could maintain the relative altitude to the landing platform while it one was oscillating in the *z*-axis. Once the landing was commanded, the RUAV reduced this distance by performing a smooth descent until it touched the landing point. During all tests, the UAV could closely follow the platform (Figure 23c) maintaining its position over the vertical of the landing point. In this case, the velocity of the moving platform reached up to 18 km/h (Figure 23d).

**Figure 24:**
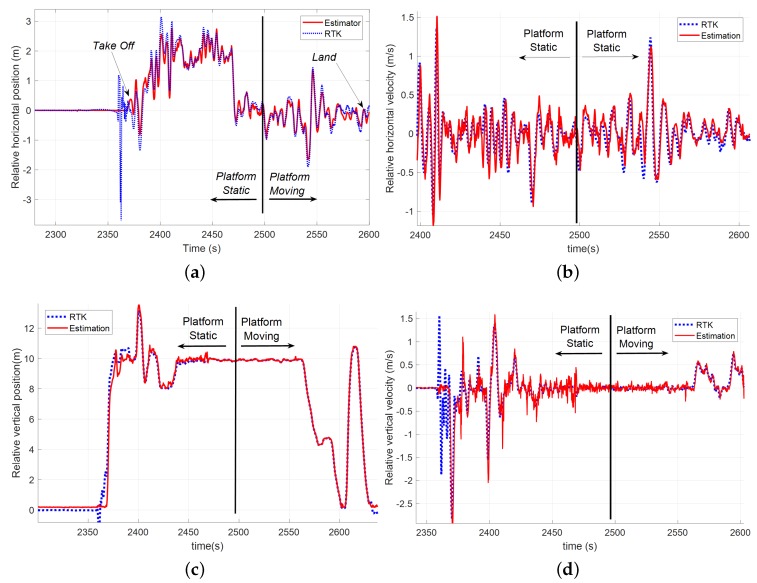
Comparison among the tether based estimation solution (red) and the GNSS-RTK system (blue) during a test where the landing platform was moving following a curvilinear trajectory: (**a**) measurements of the position in the horizontal plane; (**b**) relative velocity in the horizontal plane; (**c**) relative altitude; and (**d**) relative vertical velocity.

In this case, the test had both a static phase and a moving phase. Figure 24 shows the comparison between the tether based relative estimator and the RTK solution. As can be seen, both position and velocities estimations were very accurate and follow the dynamics of the real relative vector. In the case of the relative vertical position, the output was a little bit noisy, but it did not affect the performance of the controller. Table 4 shows the calculated errors during the static and dynamic phases of this test.

As shown in Table 4, the standard deviation and RMS error increased in the horizontal plane with respect to the straight line movement scenario. However, the accuracy was better than 20 cm and the estimations allowed the UAV to follow and land accurately in the moving platform. In this case, was also shown that the estimator module could provide very accurate estimations even in scenarios where the motion of the landing platform did not follow a straight line, an ideal condition that was imposed in the assumptions of the mathematical model of the estimation filter. These results confirm the robustness of the developed system, allowing its use in a wide spectrum of scenarios.

#### 4.2.3. Approach and High Velocity Landing

In this test, the goal was to force the helicopter to follow the moving platform at high linear velocity. The car velocity was constantly increasing with an approximate acceleration of 1.5 m/$s^{2}$ until a maximum linear velocity of approximately 10 m/s, at which the rotary wing UAV landed. In the estimation model, it was assumed that the mean relative acceleration was due solely to the helicopter dynamics, and the rest of the accelerations were treated as a zero-mean Markov process. Thus, this test allowed the evaluation of the estimator performance in scenarios where the velocity of the landing platform was increasing at a constant rate.

**Figure 25:**
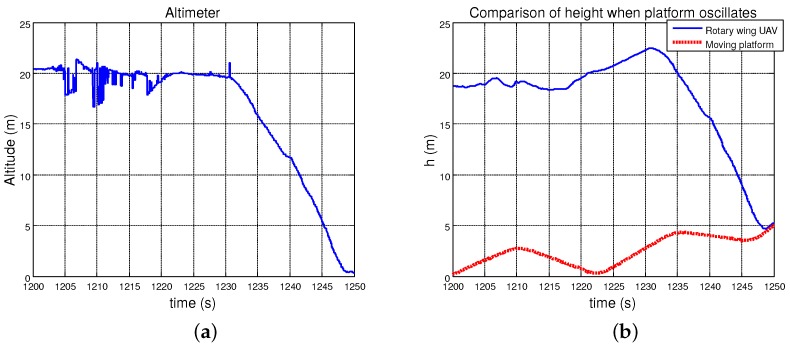

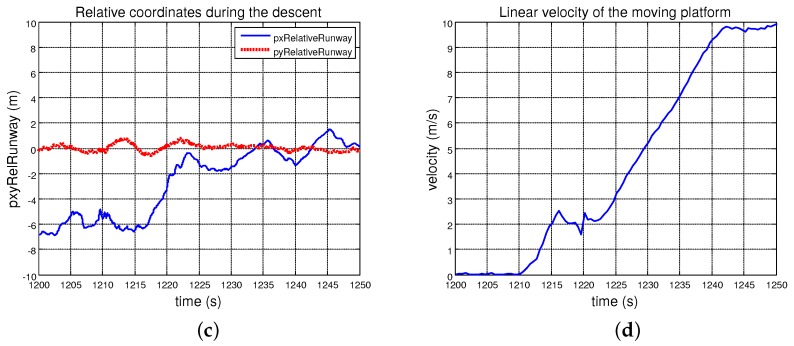
Landing maneuver data regarding high velocity test: (**a**) altimeter readings during the landing procedure, which shows the variations in the relative altitude caused by the displacement of the helicopter from a position over the ground to another over the platform; (**b**) AGL of the RUAV and the moving platform; (**c**) relative position in the horizontal axes; and (**d**) evolution of the landing platform velocity following a constant acceleration profile.

At the beginning of the maneuver, the rotary wing UAV was commanded to be 5 m behind the platform in the direction of motion in relative coordinates (see Figure 25); however, as it can be seen in Figure 25c, this distance appeared to be larger than 6 m. This error was due to the fact that the helicopter was not over the landing platform (whose dimensions are 3 m × 3 m). Hence, in this case, the altitude measured by the altimeter was relative to the ground. This caused an error in Equation (17) that propagated to the relative position measurement in Equations (21)–(23) causing the effect shown in Figure 25c. This fact suggests that, during an operation, the altimeter must only be used when the UAV is over the platform in order to avoid errors in the estimations. At *t* = 1215 s, the operator commanded the approach to the relative origin and then the UAV reached the vertical of the platform. From this moment, it can be seen in Figure 25a that the RUAV maintained the relative altitude to the landing platform by following its movement in the *z*-axis (Figure 25b). At *t* = 1231 s, the approach was commanded and the helicopter started a soft descent until it landed. During this test, the car velocity was constantly increased until a linear velocity of 35 km/h (Figure 25c), at which point the rotary wing UAV landed.

**Figure 26:**
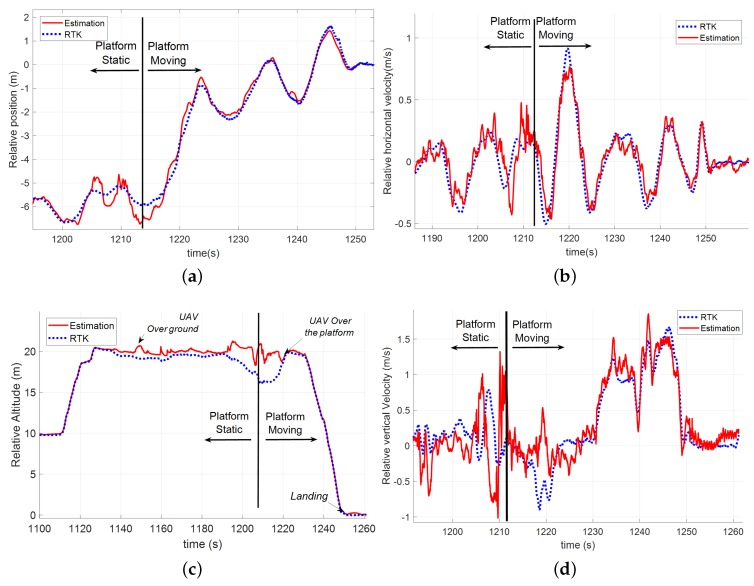
Comparison among the tether based estimation solution (red) and the GNSS-RTK (blue) measurements during a test where the landing platform was moving with a constant acceleration and reached up to 35 km/h: (**a**) position in the horizontal plane; (**b**) relative velocity in the horizontal plane; (**c**) relative altitude; and (**d**) relative vertical velocity.

As in the other dynamic scenarios, this test also included both a static phase and a moving phase. Figure 26 shows the comparison between the tether based relative estimator and the RTK solution during the test. In all the plots, it can be seen that the estimation improved at the instant in which the RUAV was over the platform (see Figure 26c). From that moment, the obtained solution was very accurate and allowed performing a safe landing by using these values as the state vector for the autopilot controller. Table 5 shows the errors in the static and dynamic phases of the test.

In this case, the relative horizontal velocity estimations were a little less accurate than those obtained during the other tests. However, the accuracy of the velocity estimation was under 0.25 m/s and was better than 30 cm in position. With these measurements, the RUAV can perform a safe landing procedure at approximately 40 km/h over a platform that is under an accelerated movement. Here, it is also interesting to note how the tether mechanism facilitated centering the UAV right on top of the expected landing position, increasing the stability of the UAV during the first instants after contacting the landing platform and helping it to remain stopped.

## 5. Conclusions and Future Work

This paper has presented an architecture based on a tether that allows a safe and accurate landing procedure of a rotary wing UAV over a moving platform. The tests presented in Section 4 demonstrated that this novel approach, with a fusion algorithm that only uses local sensors for the relative position and velocity estimation, could provide centimeter accuracy in absence of GNSS. At the same time, an acceleration model based on a time-correlated model of the acceleration with non-zero mean was implemented, which was shown to be robust against different trajectories and velocity profiles of the landing platform. This solution allowed closing the UAV control system to carry out a safe landing on static and moving platforms compensating perturbations or unmodeled behaviors. The obtained results are very promising, as they offer an alternative positioning method to GNSS allowing to land in environments with low visibility of the GNSS constellations or where the satellite signals can be jammed.

Few references about tethered UAVs can be found in the existing literature. However, many of these works describe the design of non-linear control systems and the stabilization properties that the tether provides to the aerial platform, providing mainly simulation results. Few papers provide experimental results for the tethered landing UAV maneuver, and most of them are for static landing platforms in controlled indoor scenarios. The objectives on these tests are more focused on the hovering and stabilization capabilities of the UAV than in the landing procedure. Hence, the major contributions of our paper are twofold:

- A robust estimation method based on a new tether system that provides relative measurements at 100 Hz with centimeter accuracy is presented. The system was designed, manufactured and integrated into a rotary wing UAV.
- To the best of our knowledge, these were the first field experiments with a tethered unmanned helicopter landing over a mobile platform by using the tether as its unique positioning source. A video that contains some of the tests presented in Section 4 is available at https://youtu.be/iJXoHa8EITc.

In the two last test campaigns, approximately 90% of the autonomous landing procedures were completed successfully without the intervention of the remote pilot in any phase (about 30 landings). Problems in the remaining cases were mainly due to the fact that some experiments were done with strong deceleration in the car velocity: If acceleration/deceleration occurred when the helicopter was descending to touch down and the relative altitude to the platform was very low, the helicopter might not be fast enough to compensate this acceleration/deceleration. However, in conventional operations, the dynamics of the RUAV are much faster than the landing platform’s motion, thus this issue would not represent any problem.

In the operation procedure for these tests, the helicopter took-off already attached to the platform by the tether; hence, the tether release from the helicopter and its attachment to the platform device to be rolled was not taken into account. Hence, the release of the tether from the helicopter can be a gap to be solved for real operations. The down-wash created by the helicopter could lead to undesired motion in the rope during the release.

Future developments will involve the use of a 3D LIDAR sensor in order to have another source of relative position measurements and to provide the relative attitude between the RUAV and the landing platform. This would allow testing different estimation models and to land in more complex scenarios where the pitch and roll of the landing platform can change. Finally, in a future implementation of the estimation filter, a tightly coupled architecture could be tested to check if the accuracy of the estimations can be improved by introducing the raw measurements of the sensors in the measurement model of an extended Kalman filter.

## Figures and Tables

**Figure 3:**
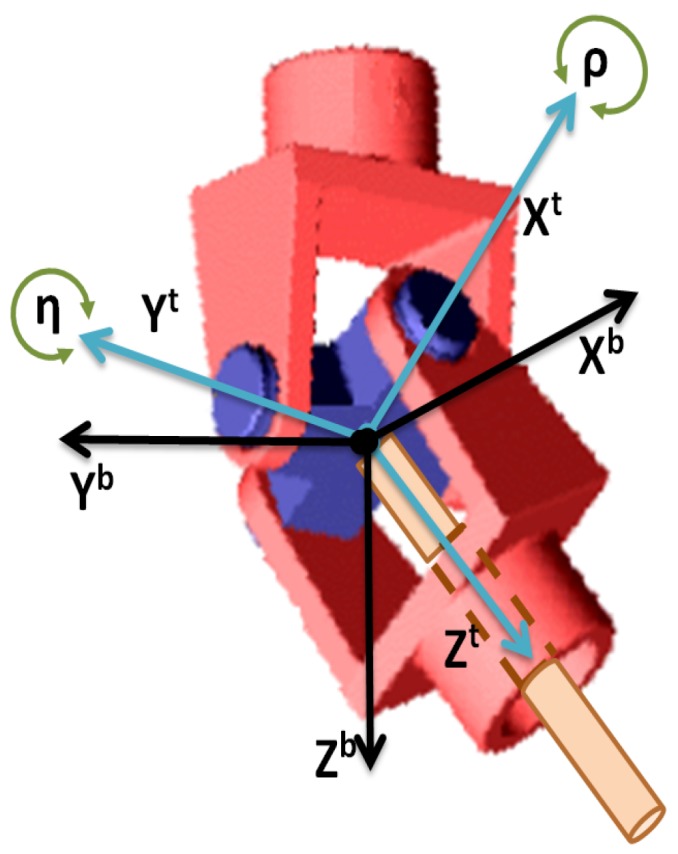
Tether device in which the tether frame *T* is represented with respect to the body frame *B*.

**Table 1:** RMS error and standard deviation of the position estimation solution (m). Calculations were performed using the RTK-GNSS system as ground truth.

Position	X North	Y West	Altitude
RMS error (m)	0.1467	0.1816	0.0043
STD	0.3451	0.3894	0.0167

**Table 2:** RMS error and standard deviation of the velocity estimation solution (m/s). Calculations were performed using the RTK-GNSS system as ground truth.

Velocity	X North	Y West	Altitude
RMS error	0.0773	0.0725	0.0557
STD	0.1971	0.1604	0.0756

**Table 3:** RMS error and standard deviation of the position (m) and velocity estimation solution (m/s) during a landing over a platform that is following a straight trajectory. Calculations were performed using the RTK-GNSS system as ground truth.

**Static Platform**	**Pos.N**	**Pos.W**	**Altitude**	**Vel.N**	**Vel.W**	**Vel.Up**
RMS error	0.045	0.052	0.107	0.040	0.059	0.049
STD	0.057	0.079	0.024	0.051	0.079	0.048
**Moving Platform**	**Pos.N**	**Pos.W**	**Altitude**	**Vel.N**	**Vel.W**	**Vel.Up**
RMS error	0.057	0.082	0.156	0.069	0.089	0.068
STD	0.061	0.074	0.148	0.061	0.108	0.091

**Table 4:** RMS error and standard deviation of the position (m) and velocity estimation (m/s) solution during a landing over a platform that was following a curvilinear trajectory. Calculations were performed using the RTK-GNSS system as ground truth.

**Static Platform**	**Pos.N**	**Pos.W**	**Altitude**	**Vel.N**	**Vel.W**	**Vel.Up**
RMS error	0.075	0.094	0.111	0.081	0.143	0.115
STD	0.088	0.119	0.153	0.105	0.191	0.161
**Moving Platform**	**Pos.N**	**Pos.W**	**Altitude**	**Vel.N**	**Vel.W**	**Vel.Up**
RMS error	0.099	0.157	0.079	0.092	0.122	0.097
STD	0.097	0.189	0.121	0.104	0.167	0.149

**Table 5:** RMS error and standard deviation of the position (m) and velocity estimation (m/s) solution during a landing over a platform that is moving at high velocity with a constant acceleration. Calculations were performed using the RTK-GNSS system as ground truth.

**Static Platform**	**Pos.N**	**Pos.W**	**Altitude**	**Vel.N**	**Vel.W**	**Vel.Up**
RMS error	0.050	0.102	0.073	0.079	0.139	0.052
STD	0.068	0.139	0.069	0.081	0.179	0.075
**Moving Platform**	**Pos.N**	**Pos.W**	**Altitude**	**Vel.N**	**Vel.W**	**Vel.Up**
RMS error	0.091	0.197	0.141	0.079	0.233	0.137
STD	0.119	0.274	0.101	0.114	0.261	0.179

**Figure A1:**
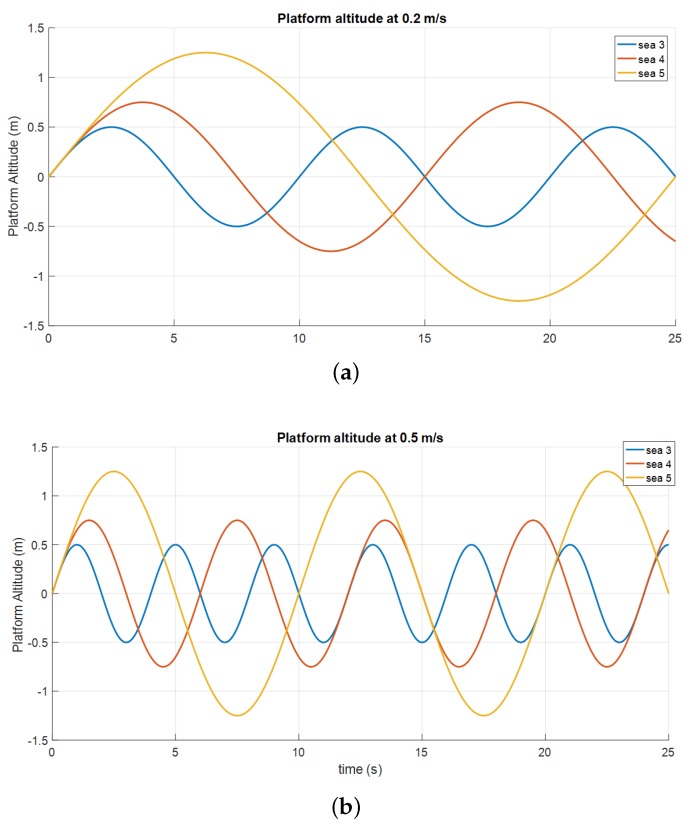
Landing platform simulating vertical ship movements under Sea 3, Sea 4 and Sea 5 at: (**a**) 0.2 m/s; and (**b**) 0.5 m/s.

**Figure A2:**
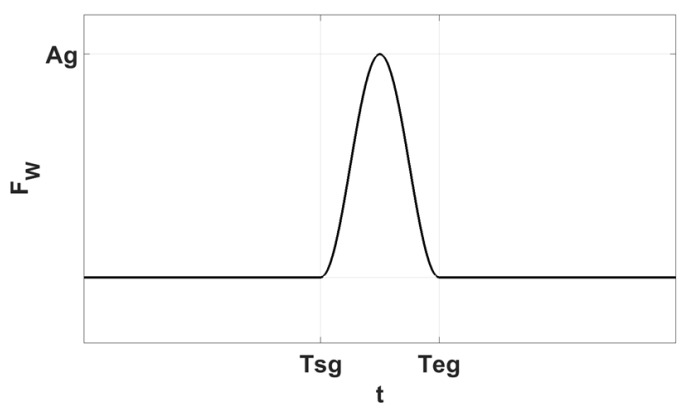
Force profile exerted by a wind gust.

**Figure A3:**
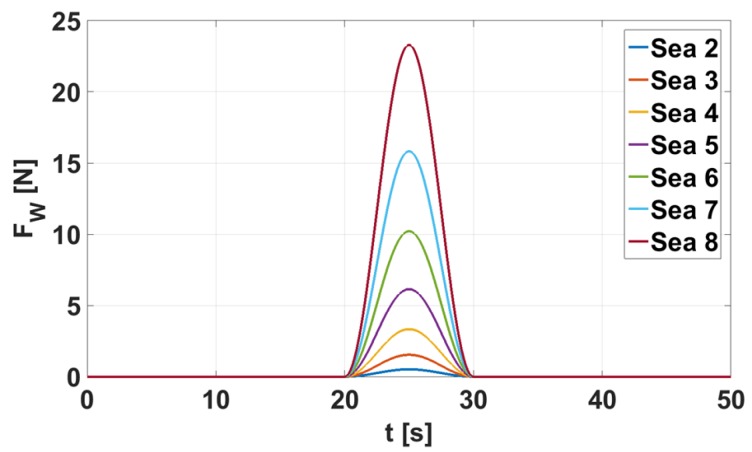
Force profiles exerted by wind gusts corresponding to different sea states.

**Figure A4:**
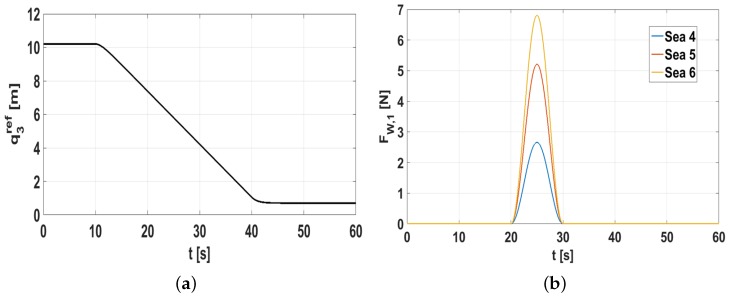
(**a**) Relative altitude reference of the helicopter; and (**b**) wind gust profiles.

**Figure A5:**
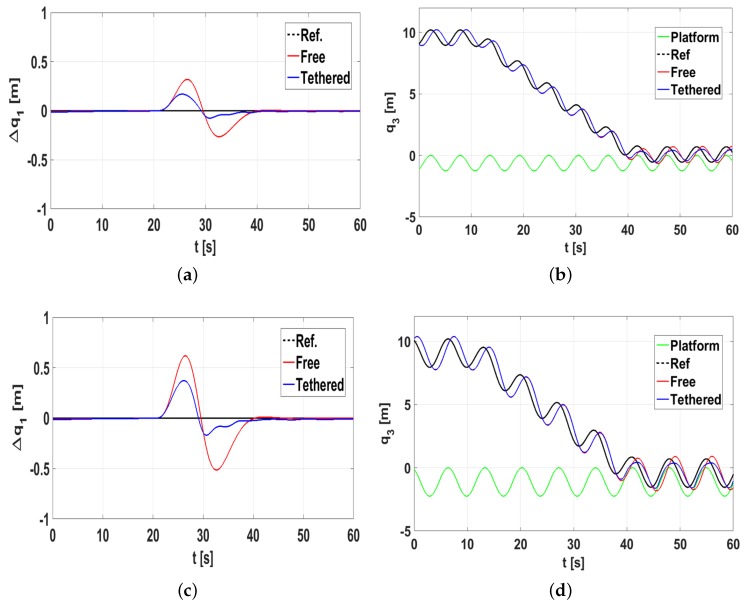

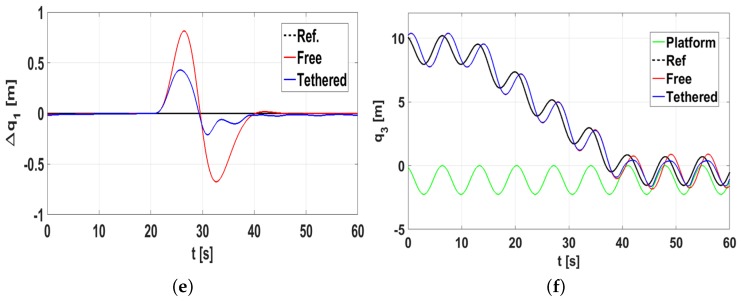
Analysis of translational dynamics between the free helicopter (red line) and the tethered approach. Helicopter longitudinal position relative to the ship deck $\Delta q_{1}$ under: (**a**) Sea State 4; (**c**) Sea State 5; and (**e**) Sea State 6. Vertical position of the helicopter and the deck $q_{3}$ under: (**b**) Sea State 4; (**d**) Sea State 5; and (**f**) Sea State 6.

## Abbreviations

The following abbreviations are used in this manuscript:

AGL	Above Ground Level
AHRS	Attitude and Heading Reference System
CP	Contact Point
GNC	Guidance, Navigation and Control
GNSS	Global Navigation Satellite System
IMU	Inertial measurement unit
INS	Inertial Navigation System
LIDAR	Light Detection and Ranging
MBZIRC	Mohamed Bin Zayed International Robotics Challenge
NWU	North-West-Up
RAST	Recovery Assist, Secure and Transverse
RC	Radio Controlled
RMS	Root Mean Square
RTK	Real Time Kinematics
RUAV	Rotary wing Unmanned Aerial Vehicle
STD	Standard Deviation
UAV	Unmanned Aerial Vehicle

## Appendix A

This appendix contains the assumptions and the constraints of the landing platform motion in order to study its similarities with the movement of a ship under different environmental conditions. Regarding the landing platform development, it is important to state that the objective was mainly to have a platform capable of performing movements in the vertical axis in order to test automated landing maneuvers in scenarios where the vehicle not only moves horizontally but also changes its position in altitude. It was not in mind to create a platform for emulating faithfully the dynamics of a ship, as this is an extremely complex task that was out of the scope of our research. However, providing to the platform of a sinusoidal movement along the vertical axis, the landing tests could be similar, in a qualitative way, to the maneuvers that would have to be carried out in a marine environment for landing on a ship’s deck.

The first limitation of the platform is that it is not possible to control the roll and pitch angles during the tests. As the platform will be towed by a car in a runway, these angles will remain almost static during the whole experiment. U.S. Department of Transportation [34] described that, when the helicopter is in contact with the surface in the landing phase, it can be affected by a lateral rolling tendency that makes the helicopter to pivot laterally around its skid, and this tendency is called dynamic rollover. It may occur during flight operations on moving vehicles if the platform is rolling and pitching while the helicopter is trying to land. This behavior can be avoided by deploying a gyro-stabilized landing platform in the vehicle allowing a safer operation by achieving a horizontally stable landing surface that minimizes the risk of entering in a dynamic rollover. Hence, the first assumption taken in this appendix is that the landing surface will be leveled during the landing operation without changes in pitch or roll.

With this assumption in mind, it is important to notice that the vertical oscillation of a ship is mainly induced by the different sea states. The sea state is the effect that the local winds have on sea conditions. The Beaufort scale is an empirical measurement that relates wind speed to observed conditions at sea. This scale has been adjusted over the past 200 years, and currently the World Meteorological Organization recognizes thirteen classes, from zero to twelve. Part of the modern Beaufort scale [35] is shown in Table A1.

**Table A1:** Beaufort scale which relates local wind with the sea state [35].

Beaufort Scale	Wind Velocity (km/h)	Wave Height (*H*) (m)	Wind Descriptive Terms
0	<1	-	Calm
1	1–5	0.1–0.2	Light air
2	6–11	0.2–0.3	Light breeze
3	12–19	0.6–1.0	Gentle breeze
4	20–28	1.0–1.5	Moderate breeze
5	29–38	2.0–2.5	Fresh breeze
6	39–49	3.0–4.0	Strong breeze
7	50–61	4.0–5.5	Near gale
8	62–74	5.5–7.5	Gale
9	75–88	7.0–10.0	Strong gale
10	89–102	9.0–12.5	Storm
11	103–117	11.5–14.0	Violent storm
12	>118	>15.0	Hurricane

The vertical oscillation of a ship deck induced by the different sea states can be approximated by the motion of the waves. To this end and according to Stewart [36], the sea-surface elevation $z_{w}$ of a wave traveling in the *x*-axis is given by

(A1) $$z_{w}=A_{w}\sin\left(\frac{2\pi }{L_{w}}x-2\pi f_{w}t\right),$$

where $A_{w}$ is the oscillation amplitude, $f_{w}$ is the wave frequency and $L_{w}$ is the wave length. The wave length is the distance between two successive wave crests. Regarding the amplitude, it is possible to relate $A_{w}$ with the Beaufort scale through the wave height *H* by

(A2) $$A_{w}=\frac{H}{2}.$$

Concerning the derivation of the wave frequency $f_{w}$, Simpson [37] established that the propagation velocity of a sea wave can be expressed as

(A3) $$v_{w}=\sqrt{\frac{gL_{w}}{2\pi }\tanh\left(\frac{2\pi D}{L_{w}}\right)}.$$

where *D* is the sea depth and *g* the acceleration due to gravity. For deep water condition in which the depth is more than half the wavelength ($\frac{D}{L_{w}}>0.5$), it is possible to approach $\tanh(\frac{2\pi D}{L_{w}})\approx 1$ resulting

(A4) $$v_{w}=\sqrt{\frac{gL_{w}}{2\pi }}.$$

Finally, using $v_{w}=L_{w}f_{w}$, the wave frequency can be written as

(A5) $$f_{w}=\sqrt{\frac{g}{2\pi L_{w}}}.$$

The former derivation allows rewriting Equation (A1) as

(A6) $$z_{w}=\frac{H}{2}\sin\left(\frac{2\pi }{L_{w}x}-\sqrt{\frac{2\pi g}{L_{w}}t}\right).$$

Combining this new expression and the Beaufort scale, the vertical oscillation of a ship deck is finally linked to the particular sea state. However, the wave properties are not independent and they should fulfill two conditions. The first condition comes from Kinsman [38]: If we can determine the wavelength, the bounds on the wave height will be given by

(A7) $$0.008L_{w}<H<0.1L_{w}.$$

The second condition can be derived from Equation (A4) and it defines the dispersion relation for deep water, quantifying the link between the period ($T_{w}$) and length of a water wave. Substituting Equation (A4) into the definition of wave speed $v_{w}=\frac{L_{w}}{T_{w}}$ yields

(A8) $$T_{w}=\frac{L_{w}}{v_{w}}=\frac{L_{w}}{\sqrt{\frac{gL_{w}}{2\pi }}}$$

(A9) $$T_{w}^{2}=\frac{L_{w}^{2}}{\left(\frac{gL_{w}}{2\pi }\right)}=\frac{2\pi L_{w}}{g}$$

(A10) $$L_{w}=\frac{g}{2\pi }T_{w}^{2};$$

where $T_{w}$ is the period of the waves (time between two consecutive waves). For the sake of simplicity, the second assumption is that the ship heave motion follows the sea surface elevation given by Equation (A6). As pointed out in Section 3, the maximum amplitude of the landing platform in vertical is 4 m and the maximum velocity is 0.5 m/s. To study the similarities between the developed platform and the vertical movement of a ship, we differentiate the following two scenarios.

**Platform is moving only in the vertical axis**: If we neglect the longitudinal motion of the ship, from Equation (A6), we could model the motion of a deck as

(A11) $$z_{w}=\frac{H}{2}\sin\left(\sqrt{\frac{2\pi g}{L_{w}}t}\right)=\frac{H}{2}\sin\left(\frac{2\pi }{T}t\right).$$

By varying the control inputs of the platform for the amplitude ($A_{cmd}$) and the vertical velocity ($v_{zcmd}$), the period of the motion can be approximated by

(A12) $$T_{p}=\frac{2A_{cmd}}{v_{zcmd}}.$$

Then, assuming that the ship follows the sea surface, and taking into the account that the maximum amplitude for the platform is 4 m and its maximum velocity is 0.5 m/s, from the dispersion relation in Equation (A10) and the boundary Equation (A7), it can be computed that the maximum sea state mechanically possible to reach is five. It is true that the 4 m altitude is inside of the Sea State 6 in the Beaufort scale, but if the platform is commanded to reach 4 m at 0.5 m/s, it will need approximately 16 s to complete a period. From the dispersion equation, this implies that $L_{w}$ should be 399 m and if we use that data in Equation (A7), we see that the condition is met.

Thus, under the assumptions done in this appendix, we can state that, if the platform is moving only in the vertical axis, it would be possible to simulate the motion of a ship from sea 0 to Sea 5. Figure A1 shows the movement of the platform when the controller is referenced with the maximum height values presented in the Beaufort table for State 3, 4 and 5 with a vertical velocity of 0.2 m/s (Figure A1a) and 0.5 m/s (Figure A1b).

**Moving platform in vertical and horizontal axes:** To emulate the motion of a ship deck during a test, the pilot of the car that tows the platform should drive following a speed profile that is related with the dispersion relation equation, the velocity of the ship, the vertical velocity of the landing platform and several more factors. Although in these tests the vertical movement of the platform did not emulate the real dynamics of a ship’s deck, from a qualitative and experimental point of view, they provide a good starting point.

## Appendix B

Due to the intrinsic risk of the landing procedure and the characteristics of the small helicopter used, tests were not carried out under wind velocities greater than 20 km/h. In this appendix, some simulations are presented to perform a comparison between the tethered helicopter configuration and a “free helicopter” by considering a wider range of sea states. The work related to the modeling and control algorithms of the helicopter and the ground tether system can be found in [19,39]. In the considered scenario of landing a helicopter on a ship’s deck, adverse weather conditions can be characterized as wind disturbances acting on the helicopter and sea waves inducing vertical oscillation on the deck, both varying its magnitude depending on the sea state. To model wind disturbances, it is required to define a proper profile for their magnitude. Additionally, it is possible to scale this profile through the empirical measurements corresponding to the different sea states using the Beaufort scale.

The wind can be modeled as a combination of different components: constant wind, ramp wind, wind gusts, etc. However, the most relevant influence concerning helicopter control in landing maneuvers is given by the wind gust. According to Ackermann [40], the gust force $F_{w}$ exerted on a helicopter can be modeled as

(A13) $$F_{w}=\left\{\begin{array}{cc} 0, & t<T_{sg}\\ \frac{A_{g}}{2}\left(1-\cos\left(2\pi \frac{t-T_{sg}}{T_{eg}-T_{sg}}\right)\right) & T_{sg}\leq t\leq T_{eg}\\ 0, & t>T_{eg} \end{array}\right.,$$

where $A_{g}$ is the gust amplitude, $T_{sg}$ is the instant when the gust starts and $T_{eg}$ is the instant when the gust finishes (see Figure A2).

According to the aerodynamics theory, the force exerted by air on a body in the flux direction, the aerodynamics drag, is given by

(A14) $$D=\frac{1}{2}\rho_{\infty }U_{\infty }^{2}A_{ref}C_{D},$$

where $\rho_{\infty }$ is the air density, $U_{\infty }$ is the relative velocity between body and air, $C_{D}$ is the drag coefficient and $A_{ref}$ is a reference area. In the proposed model for wind gusts, $U_{\infty }$ is the wind velocity (helicopter translational displacement is neglected in this landing scenario), $A_{ref}$ is the equivalent area of the helicopter fuselage and $C_{D}$ can be chosen as unit value if we consider the helicopter geometry as an equivalent cube with the same drag. Taking into account these considerations, the gust amplitude $A_{g}$ can be expressed as

(A15) $$A_{g}=\frac{1}{2}\rho_{\infty }U_{\infty }^{2}A_{ref}C_{D}.$$

Then, there is a quadratic dependence between gust amplitude and wind velocity. Table A2 shows the particular values adopted for the simulation.

**Table A2:** Modeling parameters for the gust amplitude.

	Parameter	Value	Units
Air density	$\rho_{\infty }$	1.225	kg/m${}^{3}$
Reference area	$A_{ref}$	$0.3^{2}$	m${}^{2}$
Drag coefficient	$C_{D}$	1	-

By using the Beaufort scale presented in Table A1 and Equations (A13)–(A15), the force exerted by a wind gust can be modeled for the different sea state conditions. In Figure A3, the force values calculated using the maximum values of the Beaufort scale have been applied together with a gust duration of 10 s, a representative value according to Amaya et al. [41].

This analysis is based on several simulations that compare the performance of the tethered configuration with the performance of the free helicopter. The simulation, similar to the experiments, consisted of two phases: in the first phase, the tethered helicopter tried to keep its vertical position with respect to a platform with forward motion and vertical oscillations; whereas the second phase corresponded to the helicopter landing maneuver on the platform.

In these simulations, the ship deck was moving forward with a constant speed of 1 m/s and it was vertically oscillating according to the sea state characterization presented in Appendix A. It was also assumed that a stabilized platform is mounted on the ship deck. Regarding the helicopter, it started the landing maneuver at *t* = 10 s. Additionally, a wind gust was added in the longitudinal direction, again according to the sea state characterization. Furthermore, for comparative purposes along different sea state conditions, the simulations were carried out for wind gusts and ship vertical oscillations equivalent to Sea States 4–6 (Beaufort scale). The behavior of the helicopter in free flight trying to perform the same landing maneuver was considered to underline the benefits of the tether configuration in this particular scenario.

Figure A4 shows the relative altitude reference commanded to the RUAV during the simulation ($q_{3}^{ref}$) and the longitudinal wind gust forces ($F_{W,1}$) used between Seconds 20 and 30.

Figure shows the variables directly affected by the disturbances, the helicopter longitudinal position relative to the ship deck $\Delta q_{1}$ (highlighting in the graph the improvement percentage of the tethered helicopter with respect to the free helicopter), and the vertical position of the helicopter and the deck $q_{3}$.

In general, the tethered configuration exhibited better performance than the free helicopter. It can be seen in the longitudinal direction, where the wind gusts were present and the ship was moving, since the tethered configuration always overcame the free helicopter due to the tether tension stabilizing effect. Regarding the vertical direction, it was noticeable that the free helicopter was not able to keep its relative position to the ship deck for the sea states considered here. When analyzing the tether approach that succeeded in maintaining such relative position, it could be found that the tracking capability decreased as the sea state increased (as expected).
